# Long-Term Treatment with *Alcaligenes faecalis* A12C Improves Host Resistance to Pathogens in Septic Rats: Possible Contribution of Curdlan-Like Immune Trainer

**DOI:** 10.1007/s12602-024-10252-0

**Published:** 2024-04-26

**Authors:** C. J. Martel-Benítez, R. Alayón-Afonso, D. Padilla Castillo, F. J. Chamizo-López, M. Isabel García-Laorden, A. Espinosa de los Monteros y Zayas, J. C. Rivero-Vera, P. Nogueira Salgueiro, F. Real, A. Bordes-Benítez, A. Martel Quintana, C. Almeida Peña, C. Domínguez Cabrera, J. M. González-Martín, J. Martín Caballero, R. Frías Beneyto, Jesús Villar, J. L. Martín-Barrasa

**Affiliations:** 1 Group of Fish health for aquaculture and wild species, Infectious diseases and Food safety, University Institute of Animal Health and Food Safety (IUSA), University of Las Palmas de Gran Canaria, 35416 Arucas, Spain; 2 Microbiology Department, Hospital Universitario de Gran Canaria Dr. Negrín, Barranco de La Ballena s/n, 35019 Las Palmas de Gran Canaria, Spain; 3 CIBER de Enfermedades Respiratorias, Instituto de Salud Carlos III, Monforte de Lemos 3-5, Pabellón 11, 28029 Madrid, Spain; 4 Multidisciplinary Organ Dysfunction Evaluation Research Network, Research Unit, Hospital Universitario de Gran Canaria Dr. Negrín, Barranco de La Ballena s/n, 35019 Las Palmas de Gran Canaria, Spain; 5 Morphology Department, University Institute of Animal Health and Food Safety (IUSA), Universidad de Las Palmas de Gran Canaria, Arucas, Las Palmas, Spain; 6 Pathology Service, Hospital Universitario de Gran Canaria Dr. Negrín, Barranco de La Ballena S/N, 35019 Las Palmas de Gran Canaria, Spain; 7 Clinical Biochemistry Department, Hospital Universitario de Gran Canaria Dr. Negrín, Barranco de La Ballena s/n, 35019 Las Palmas de Gran Canaria, Spain; 8 Banco Español de Algas, Instituto de Oceanografía y Cambio Global, Universidad de Las Palmas de Gran Canaria, Telde, Spain; 9 Banco Español de Algas, Fundación Parque Científico Tecnológico, Universidad de Las Palmas de Gran Canaria, Telde, Spain; 10 Statistics Service, Research Unit, Hospital Universitario de Gran Canaria Dr. Negrín, Barranco de La Ballena s/n, 35019 Las Palmas de Gran Canaria, Spain; 11 Barcelona, Biomedical Research Park (PRBB), Barcelona, Spain; 12 Deparment of Laboratory Medicine and Comparative Medicine, Karolinska Institutet, Stockholm, Sweden; 13 Fundación Canaria del Instituto de Investigación Sanitaria de Canarias (FIISC), Las Palmas de Gran Canaria, Spain; 14 Animal Facility, Research Unit, Hospital Universitario de Gran Canaria Dr. Negrín, Barranco de La Ballena s/n, 35019 Las Palmas de Gran Canaria, Spain; 15 CIBER de Enfermedades Infecciosas (CIBERINFEC), Instituto de Salud Carlos III, Madrid, Spain

**Keywords:** *Alcaligenes faecalis*, Sepsis, Probiotic, Curdlan, Rat, Cytokines

## Abstract

Curdlan, a β-1,3/1,6-glucan found in *Alcaligenes faecalis* (*A. faecalis*) wall, activates innate and humoral immunity. The aim of this study is to evaluate whether pretreated rats with *A. faecalis* A12C could prevent sepsis disturbances and identify the immunomodulatory mechanisms involved. Experiments occurred in two stages: a survival study with 16 rats randomly divided into septic (SC) (*n* = 8) and septic pretreated (SA) (*n* = 8) groups and 45 rats divided into four groups: healthy (AGUSAN) (*n* = 9), septic (AGUIC) (*n* = 13), septic pretreated (AGUIA) (*n* = 14), and healthy pretreated (AGUSTO) (*n* = 9). Sepsis was induced by cecal ligation and puncture after 30 days of *A. faecalis* A12C pretreatment or without. SA group had a higher survival rate of 58% vs. 16% for SC group (*P* < 0.05). Overall, AGUIA showed better status than AGUIC (*P* < 0.01). Higher monocytosis was found in AGUIA and AGUSTO vs. AGUIC and AGUSAN, respectively (*P* < 0.05). A gradual increase in curdlan fecal concentration was observed in AGUIA during pretreatment. Fecal concentrations of *Escherichia coli* significantly decreased in AGUIA and AGUSTO. Bacterial load in urine, peritoneal lavage fluid (PLF), and bronchoalveolar lavage fluid (BALF) decreased (*P* < 0.05) in AGUIA vs. AGUIC. Finally, lower inflammation was observed in serum, BALF, and PLF, with reduced IL-6, IL-10, IL-1β, and TNF-α, along with less damage in lungs and peritoneum in AGUIA vs. AGUIC. These findings suggest the connection between curdlan—produced by *A. faecalis* A12C—with the immune system and the reduction in severity of experimental sepsis.

**Supplementary Information:**

The online version contains supplementary material available at 10.1007/s12602-024-10252-0.

## Introduction

In recent years, there has been an increase in the number of studies associating the use of bacterial strains with health benefits, including *Bifidobacterium* spp., *Lactobacillus* spp., *Enterococcus* spp., *Streptococcus* spp., or *Bacillus* spp. These probiotic candidates have shown benefits on human and/or animal health [1–3]. Some authors have explained the beneficial effects based on the ability to promote the production of anti-inflammatory cytokines or to inhibit pro-inflammatory cytokines [4].

While lactic acid bacteria have traditionally been the most commonly employed probiotics in the fields of human and veterinary medicine, there is a growing trend in research focused on the assessment of novel bacterial species that may have potential utility as probiotics [5–9]. In several studies by our group, a strain of *Alcaligenes faecalis* subsp. *faecalis* (*A. faecalis* A12C), isolated from *Argyrosomus regius* gills, has shown excellent conditions as potential probiotic in fish due to inhibitory activity against different pathogens, particularly *Photobacterium damselae* subsp. *piscicida* and *Vibrio anguillarum*, stimulating their immune response by activating a variety of pro-inflammatory cytokines, such as interleukin-6 (IL-6) and tumor necrosis factor alpha (TNF-α) [10, 11]. Our group reported the first study examining the probiotic effects of *A. faecalis* A12C on general, biochemical, and pathological parameters in a clinically relevant rodent model of peritonitis, showing that oral administration of this strain during 7 days is safe and had marked effects on the spread of infection [12].

**Fig. 1:**
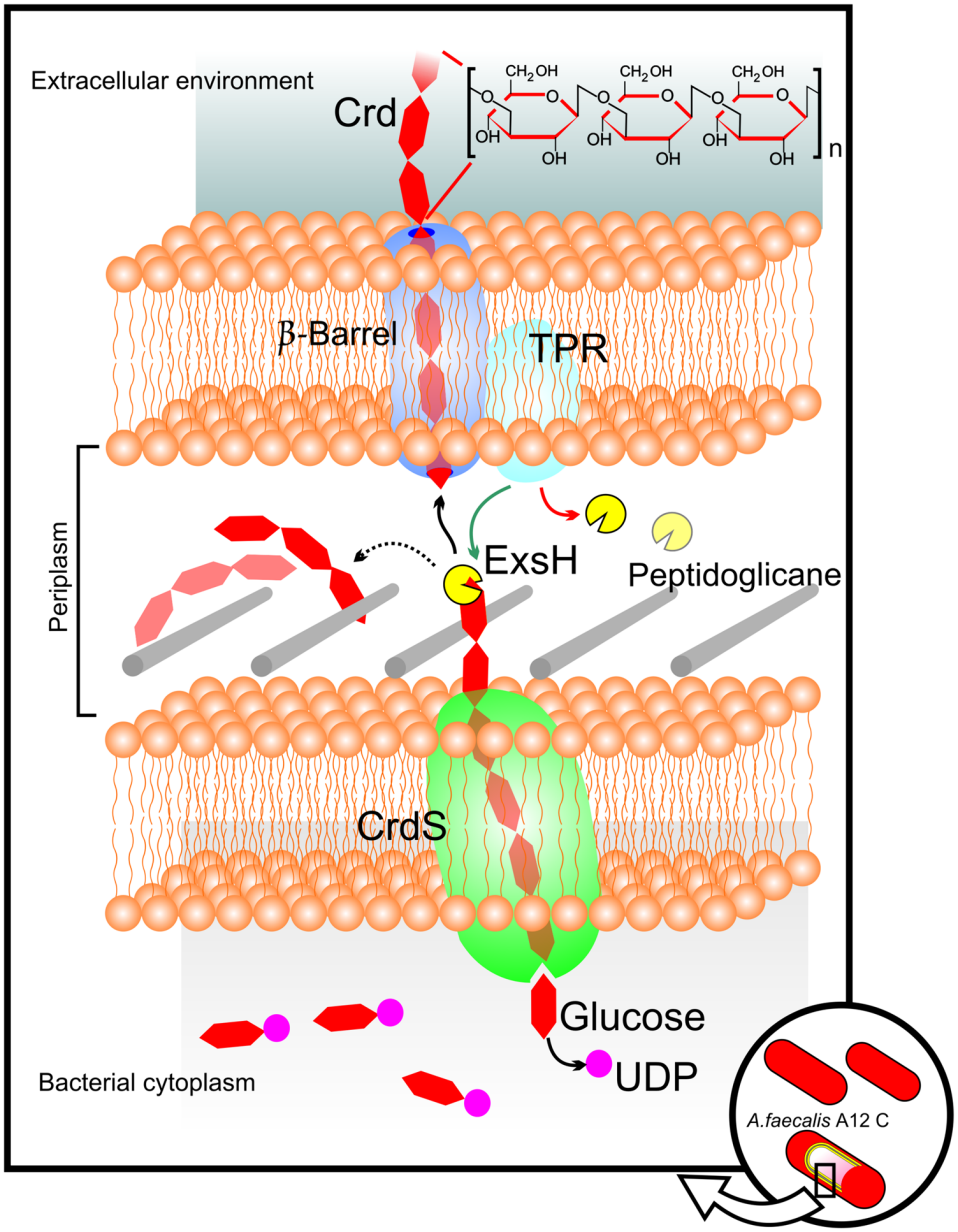
Diagram of the curdlan biosynthesis and secretion system in *A. faecalis*

*A. faecalis* has aerobic growth at 37 °C and at a neutral pH in blood agar, producing a large amount of curdlan when there is a limitation of nitrogen and a supply of ammonium phosphate in the culture media [5, 7]. Curdlan is a linear polymer composed of a β-1,3-glucan, whose only basic unit is glucose with a high molecular weight [13]. The main β-glucan molecules are found in the cell wall of organisms such as bacteria, yeasts, fungi, algae, and plants. In *A. faecalis*, curdlan is polymerized by the β-1,3-glucan synthase CrdS in the inner membrane. It is speculated to provide a channel for the export of the high-molecular-weight curdlan chain [14]. After entering the periplasm, the polysaccharide is hydrolyzed by the β-endonuclease ExsH and crosses the outer membrane passing through a β-barrel membrane protein. A tetrapeptide repeat (TPR) protein protects the glycan chain during secretion and regulates the degradation of polysaccharide chains by the β-endonuclease ExsH [15] (Fig. 1).

This polysaccharide activates the innate immune system and humoral immunity [15, 16], providing protection against otherwise severe or lethal infections. There is extensive scientific evidence about the protective effect of β-glucans against bacterial and fungal infections [17, 18] and viral and parasitic infections [19–21] through immunomodulating mechanisms. The glucans were able to increase secretion of interleukins, increase expression of dectin-1 on macrophages, and increase expression of Toll-like receptor 2 on dendritic cells [22].

Based on this background, we decided to test whether *A. faecalis* A12C can provide protection against a clinically relevant model of sepsis in vivo. We evaluated the immunomodulatory effect of *A. faecalis* A12C on certain cytokines and the production of curdlan by this bacterial strain.

## Materials and Methods

### Animals

In accordance with the guidelines outlined by the European Commission (2010/63/EU) and Spanish Legislation (Law 53/2013) for the protection of animals in scientific research, this study received approval from the Local Animal Ethics Committee at the Hospital Universitario de Gran Canaria Dr. Negrín (HUGCDN) in Las Palmas de Gran Canaria, Spain.

A total of 61 male pathogen-free Crl Sprague Dawley® (SD) rats, each 12 weeks old, were employed for this study. These rats were maintained under semi-barrier conditions at the animal facilities of HUGCDN. The rats were housed in pairs and provided with enrichment items such as igloos and nesting material. Cage changes were carried out twice a week. The rats had access to a diet comprising rat chow pellets (Teklad® Global 14% Protein Rodent Maintenance Diet, Harlan, Barcelona, Spain) and ad libitum access to drinking water (Fonteide®, S/C de Tenerife, Spain). The lighting conditions followed a 12:12-h light/dark cycle. Room temperature was maintained at 21 ± 1 °C, and relative humidity was maintained at 55 ± 5%, with an air exchange rate of 15 times per hour (details in Supplementary Material and Methods).

### Preparation of A. faecalis A12C

The probiotic strain *A. faecalis* A12C was isolated from *Argyrosomus regius* gills at the Instituto de Sanidad Animal y Seguridad Alimentaria of the University of Las Palmas de Gran Canaria, Spain. Fresh cultures were made as previously described by our group [12] (details in Supplementary Material and Methods).

### Sepsis Model

Sepsis was induced by cecal ligation and puncture (CLP) method as previously described [23]. All procedures were performed under general anesthesia, with a subcutaneous cocktail of fentanyl (Fentanest®, Kern Pharma, Barcelona, Spain) and medetomidine (Domtor®, Orion, Espoo, Finland), both at 0.3 mg/kg. Briefly, after a laparotomy, the cecum was ligated at half the distance between distal pole and the base of the cecum to induce mild-grade sepsis and perforated twice; feces were extruded, and the abdominal incision was closed. Animals received prewarmed normal saline (37 °C; 5 ml per 100 g body weight) subcutaneously for fluid resuscitation. For postoperative analgesia, all animals’ welfares were evaluated (Supplementary Table 1) [24] and treated subcutaneously with buprenorphine (Buprex®, Indivior Europe Ltd, Dublin, Ireland) 0.05 mg/kg for at least 2 days (details in Supplementary Material and Methods).

### Experimental Design

This study was conducted in two phases. In the first phase, 16 animals were randomly divided into two groups for examining survival: (i) septic control group (SC) (*n* = 8), in which *A. faecalis* A12C was not administered and sepsis was induced by CLP, and (ii) septic group pretreated with *A. faecalis* A12C (SA) (*n* = 8), in which the probiotic was administered during 30 days in the drinking water (6 × 10^8^ CFU/ml) and water were replenished daily. In a second phase, 45 experimental animals were randomly divided into four groups: (i) healthy control group (AGUSAN) (*n* = 9), in which *A. faecalis* A12C was not administered nor was subjected to sepsis; (ii) septic control group (AGUIC) (*n* = 13), in which *A. faecalis* A12C was not administered and sepsis was induced by CLP; (iii) septic group pretreated with *A. faecalis* A12C (AGUIA) (*n* = 14), in which the probiotic was administered during 30 days in the drinking water; and (iv) healthy pretreated control group (AGUSTO) (*n* = 9), in which *A. faecalis* A12C was administered during 30 days in the drinking water but CLP was not performed.

**Fig. 2:**
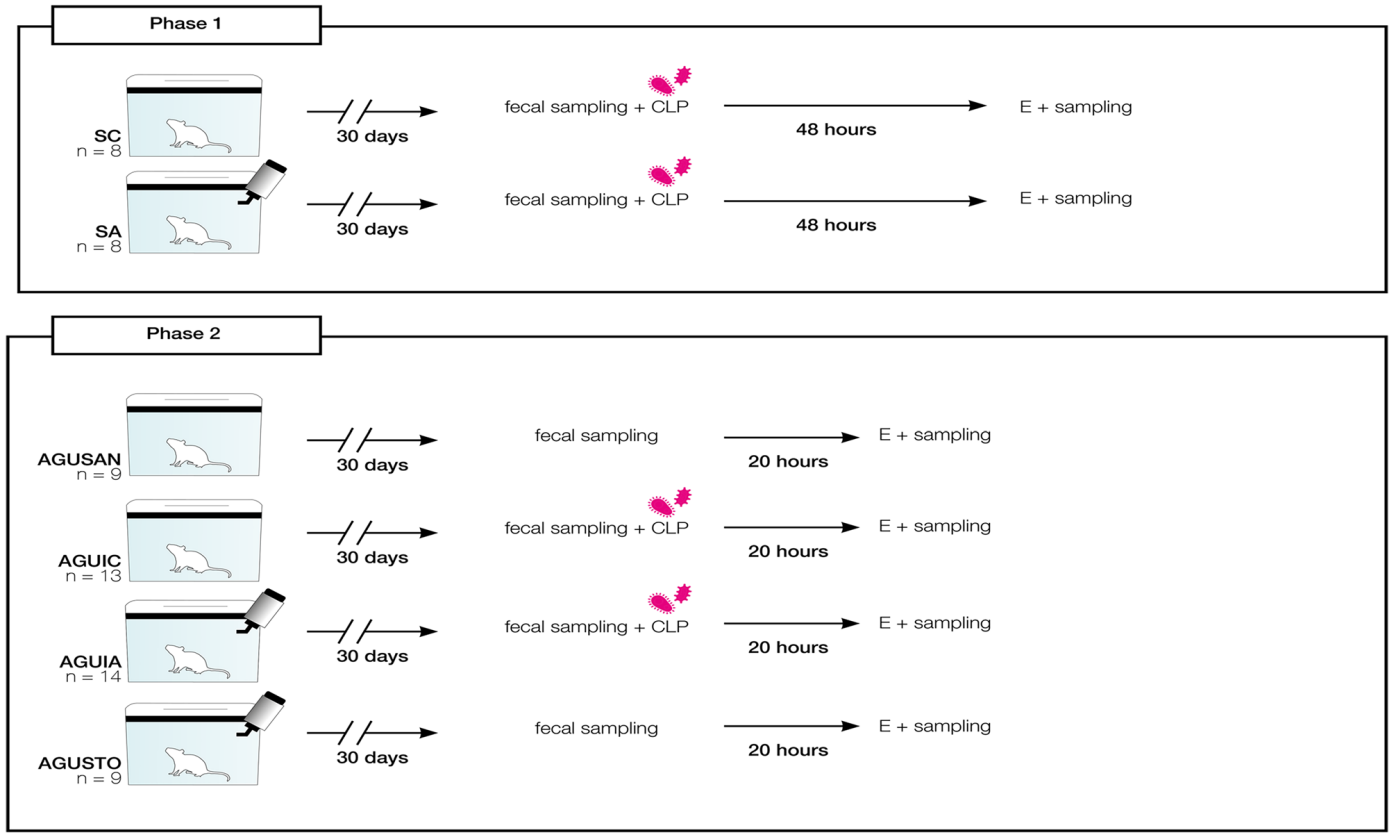
Experimental design and timeline. Phase 1, survival study: septic group without *A. faecalis* A12C (SC), and septic group but pretreated with *A. faecalis* A12C (SA) during 30 days in the drinking water (6 × 108 CFU/ml). Phase 2, healthy control group (AGUSAN) without *A. faecalis* A12C; septic control group induced by CLP, without *A. faecalis* A12C (AGUIC); septic group pretreated with *A. faecalis* A12C (AGUIA), in which the probiotic was administered during 30 days in the drinking water (6 × 10^8^ CFU/ml); and healthy control group (AGUSTO), in which *A. faecalis* A12C was administered during 30 days in the drinking water (6 × 10^8^ CFU/ml). CLP, cecal ligation and puncture method; E, euthanatized

After 30 days of probiotics or water alone, a hematological study and microbiological examination of the feces were made in all animals. Surviving rats were euthanized, and samples were collected at 48 h after CLP in SC and SA (phase 1), at 20 h post-CLP in AGUIC and AGUIA, and at 20 h after 30 days of probiotics or water administration in AGUSTO and AGUSAN groups (phase 2). Figure 2 summarizes the experimental design.

### Body Temperature, Weight, and Sample Collection

At the end of each experimental phase, animals were weighed (body temperature was measured) and anesthetized. In anesthetized animals, the following samples were collected: blood from external jugular vein for blood count and biochemistry, intracardiac blood for blood culture and serum collection, urine by direct puncture of urinary bladder for urine culture, bronchoalveolar lavage fluid (BALF) from the right lung (2–3 ml), and peritoneal lavage fluid (PLF) from the abdominal cavity (4 ml) for microbiological culture, cytological analysis, and cytokine and curdlan measurements. Finally, liver, kidney, spleen, duodenum, pancreas, jejunum, mesenteric lymph nodes, thymus, and lung were collected for histological evaluation. Rats were euthanized by exsanguination after excision of the caudal vena cava and abdominal aorta.

### Blood Count and Blood Chemistry

Whole blood samples were analyzed to determine hematological parameters, including hematocrit, red blood cell count, platelet count, total white blood cell count, lymphocytes, mononuclear cells, neutrophils, eosinophils, and basophils. Additionally, serum biochemical parameters such as alanine aminotransferase (ALT), aspartate aminotransferase (AST), creatinine (CREA), urea (UREA), and C-reactive protein (CRP) were quantified (details in Supplementary Material and Methods).

### Bacterial Counts in Feces

To assess the gut survival capabilities of the probiotic *A. faecalis* A12C and its impact on *E. coli* growth within the fecal microbiota, the bacterial load was quantified in colony-forming units per gram (CFU/g) for both bacterial species. Fecal samples were obtained from the animals by gently massaging the abdomen at the beginning and at the end of each experimental phase. To regulate bacterial abundance, tenfold dilutions were prepared in 0.9% sterile saline and plated on agar plates containing 4.5 g/100 ml sodium chloride, 64 mg/l vancomycin (SALA, Barcelona, Spain), and 0.02 g/l bromothymol blue (Merck, Darmstadt, Germany). Incubation was carried out at 37 °C for 24 h (for *E. coli*) or 72 h (for *A. faecalis* A12C) to calculate the total number of viable bacteria (CFU/g of feces) in the original sample. Colonies were isolated and subjected to repeated subcultures. Bacterial species were identified based on colony morphology and Gram staining. Advanced identification was performed using the MALDI-TOF MS (Vitek® MS, Biomerieux) technique [25].

### Microbiological Analysis

Samples were processed within 2 h of collection. Both aerobic and anaerobic microorganisms were assessed from PLF, BALF, and urine using 100 µl of the sample and 3 ml of blood sample. Incubation conditions were maintained at 37 °C. Aerobic microorganisms were cultured in 10 ml Brain–Heart Infusion broth (PanReac AppliChem), and after 24 h, 25 µl of these cultures was streaked onto various agar media, including CLED, MacConkey, Mannitol Salt Agar, and Sabouraud Dextrose Agar (all supplied by PanReac AppliChem). Anaerobic microorganisms were plated on Brucella Blood Agar with Hemin and Vitamin K1 (Becton Dickinson) and incubated for 4 days under anaerobic conditions. To investigate aerobic and anaerobic bacteremia, blood samples were inoculated into 10 ml of BD BACTEC Lytic Anaerobic (Becton Dickinson) and placed in the BD BACTEC FX blood culture system (Becton Dickinson) for 5 days. If any signs of bacterial growth were observed, a secondary culture was prepared on Blood Chocolate and MacConkey agar (both from Becton Dickinson), and the plates were incubated for 48 h in a 5% CO_2_ environment. Additionally, Brucella Blood Agar with Hemin and Vitamin K1 (Becton Dickinson) was incubated for 4 days under anaerobic conditions. Bacterial species were identified based on colony morphology and Gram staining, and advanced identification was carried out using the MALDI-TOF MS (Vitek® MS, Biomerieux) technique.

### Cytokine Assays

Serum, BALF, and PLF concentrations of IL-1β, IL-6, IL-10, TNF-α, and interferon (IFN)-γ were determined in duplicates using available ELISA kits for rat cytokines (details in Supplementary Material and Methods).

### Curdlan Measurement

In all groups of animals of the second phase of the study, curdlan concentrations in feces, urine, serum, PLF, and BALF were measured (details in Supplementary Material and Methods). Curdlan concentrations in feces were assessed at 0 day, 7 days, 15 days, and 30 days after the first dose of *A. faecalis* A12C. Urine, serum, PLF, and BALF curdlan concentrations were assessed at 30 days after the first dose of *A. faecalis* A12C. Before analysis, the samples used were previously lyophilized and the concentrations were determined as percentage of curdlan in the dry weight of the samples.

### PLF, BALF, and Urine Cytology

Immediately after sampling, the PLF, BALF, and urine specimens were equally divided into three 200 µl K3-EDTA microtubes. Samples were analyzed with a flow cytometer using setting for rats [26, 27] for reporting differential count of total nucleated cells (TNCC), granulocytes (GRAN), agranulocytes (AGRAN), and red blood cells (RBC) (details in Supplementary Material and Methods).

### Histological Evaluation and Wet-to-Dry Lung Weight Ratio

Tissue samples from animals of the second experimental phase were fixed in 4% formalin for 24 h, embedded in paraffin, and cut into 4-µ sections for histological study. Slides were subjected to staining with hematoxylin–eosin and subsequently scrutinized using a light microscope. Slides were evaluated by two pathologists blinded to experimental groups (details in Supplementary Material and Methods). In all organs, an assessment was made regarding the presence of bacteria within the vessels, which involved microscopic observation of tiny rod-shaped structures exhibiting a blue hue inside these vessels. Additionally, evaluations were conducted for parenchymal disarray, interstitial swelling, the infiltration of white blood cells, tissue damage, and interstitial bleeding. Lung damage was quantified using a semi-quantitative scoring system that considered factors such as thickening of alveolar septa, the presence of neutrophils in the interstitial or alveolar areas, the formation of hyaline membranes, and the accumulation of proteinaceous material within the airspaces [28]. Heart injury was assessed based on the presence of an inflammatory response in the epicardium and/or myocardium. Evaluation of intestinal damage encompassed examinations at both mucosal and serosal levels. Our histological indicators for renal damage included the vacuolization of proximal tubule cells, loss of the brush border, and shedding of cells into the tubular lumen. Hepatic sections were examined for decreased staining, vesicular nuclei, and opening of the sinusoids [29]. Peritoneal injury was assessed based on a semi-quantitative severity score of histopathological peritonitis [30] as 0, 1, 2, and 3.

Wet-to-dry (W/D) lung weight ratio was evaluated. Left lungs were dissected free of hilar structures, weighed (wet weight), dried at a constant temperature of 50 °C in a gravity convection oven (Memmert GmbH, Schwabach, Germany) for 72 h, and weighed again (dry weight) to obtain the W/D lung weight ratio as a marker of extravascular lung water [31].

### Statistical Analysis

The analyses were conducted using the Statistical Package R 2019 version 3.5.3 (R Foundation for Statistical Computing, Vienna, Austria). To assess the normality of quantitative variables, the Shapiro–Wilk test was utilized. Continuous variables are presented as medians along with the 25th and 75th percentiles. When making comparisons among more than two groups, the Kruskal–Wallis test was employed, and adjustments for multiple comparisons were made using the Bonferroni correction as necessary. Comparisons of two independent groups were conducted using the Mann–Whitney *U* test. Survival rate studies were analyzed using Kaplan–Meier methods, followed by log-rank tests. Data are visually represented using box and whisker plots. In all cases, two-tailed tests were performed, and statistical significance was considered achieved at a significance level (*P* < 0.05).

## Results

### Evaluation and Survival

**Fig. 3:**
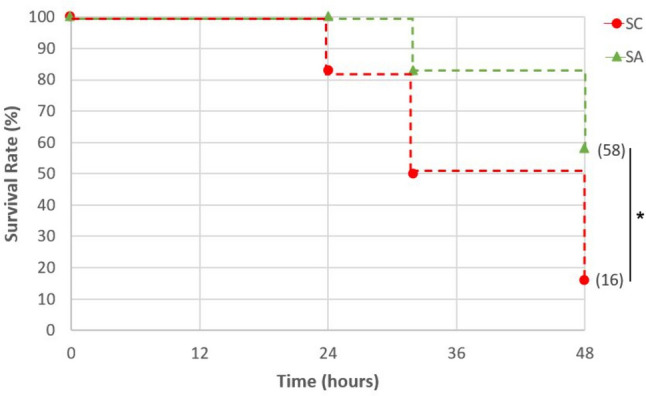
Survival time after CLP. Septic control group (SC) vs. septic pretreated with *A. faecalis* A12C during 30 days group (SA). *P < 0.05

**Fig. 4:**
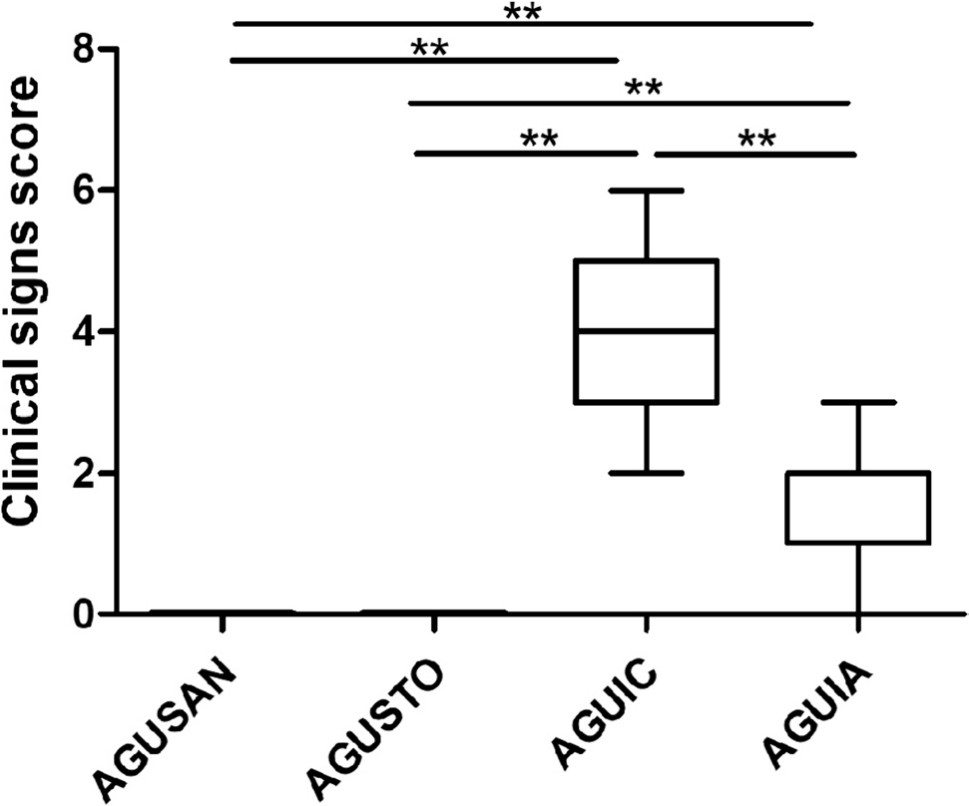
Level of discomfort in healthy groups (AGUSAN and AGUSTO) and septic groups (AGUIC and AGUIA) just before euthanasia, according to the clinical signs shown in Supplementary Table 1. Box and whisker diagrams depict the smallest observation, lower quartile, median, upper quartile, and largest observation. **P < 0.01

In the first phase, to investigate the effects of *A. faecalis* A12C on the survival rate of septic rats, survival rate was measured at 0 h, 12 h, 24 h, 36 h, and 48 h after CLP. Overall survival rate of septic group pretreated with *A. faecalis* A12C (SA) was higher than that of those untreated (58% vs. 16%, *P* < 0.05) (Fig. 3). In the second phase, survival at 20 h after CLP was 100% in AGUIC and AGUIA, as in their healthy control groups (AGUSAN and AGUSTO). However, differences in clinical signs were observed (Fig. 4). Greater discomfort was found in the group of septic animals not treated with probiotics (AGUIC) compared to septic ones and pretreated with A12C (AGUIA). The most frequent clinical signs present were ruffled fur, chromodacryorrhea, and dehydration. There were no significant differences between AGUSAN and AGUSTO. At the end of the second phase, all experimental groups did not show significant changes in body weight (Supplementary Fig. 1).

### Blood Count and Blood Chemistry

**Fig. 5:**
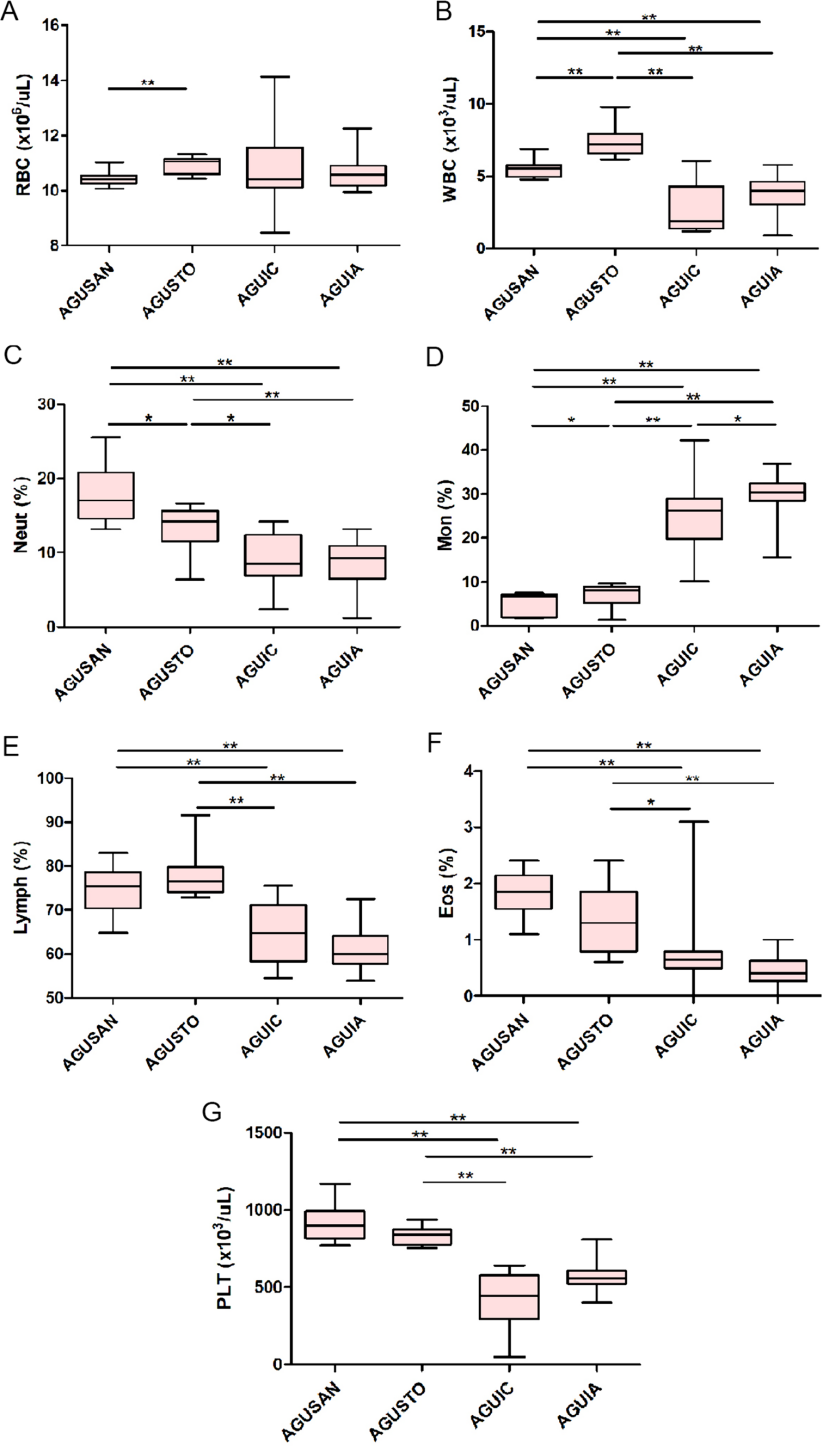
Hematological parameters in healthy groups (AGUSAN and AGUSTO) and septic groups (AGUIC and AGUIA) at the time of euthanasia (phase 2). **A** RBC, red blood cells; **B** WBC, white blood cells; **C** Neut, neutrophils; **D** Mon, monocytes; **E** Lymph, lymphocytes; **F** Eos, eosinophils; **G** PLT, platelets. Box and whisker diagrams depict the smallest observation, lower quartile, median, upper quartile, and largest observation. *P < 0.05; **P < 0.01

In regard to hematological parameters from phase 2 (Supplementary Table 2), in most cases, septic animals pretreated with *A. faecalis* A12C (AGUIA) had closer values to healthy pretreated animals (AGUSTO) or non-pretreated (AGUSAN) with *A. faecalis* A12C, than the septic group without pretreatment with *A. faecalis* A12C (AGUIC). An increase in RBC concentration (*P* < 0.01) was observed in AGUSTO group compared to AGUSAN group (11.05 × 10^6^/µl vs. 10.41 × 10^6^/µl, respectively) (Fig. 5). Thrombocytopenia was also found in septic animals when compared to non-septic animals (*P* < 0.01). However, a decrease in platelets was much lower in animals pretreated with *A. faecalis* A12C than in non-pretreated septic groups (558.5 × 10^3^/µl vs. 442.5 × 10^3^/µl) (Supplementary Table 2). AGUIC and AGUIA septic groups had marked leukopenia (*P* < 0.01) compared to non-septic groups, although leukopenia was lower in AGUIA group (4.02 × 10^3^/µl) than in AGUIC (1.89 × 10^3^/µl). In addition, a significant increase (*P* < 0.01), within physiological ranges, in the concentration of leukocytes was observed in the AGUSTO group (7.22 × 10^3^/µl) compared to the AGUSAN group (5.54 × 10^3^/µl). Finally, in both septic groups (AGUIC and AGUIA), significant decreases (*P* < 0.05) were observed in the percentages of lymphocytes, eosinophils, and neutrophils. On the other hand, monocytosis was found in the septic groups, which was more pronounced in AGUIA (30.4%) than in AGUIC (26.4%) (*P* < 0.05).

**Fig. 6:**
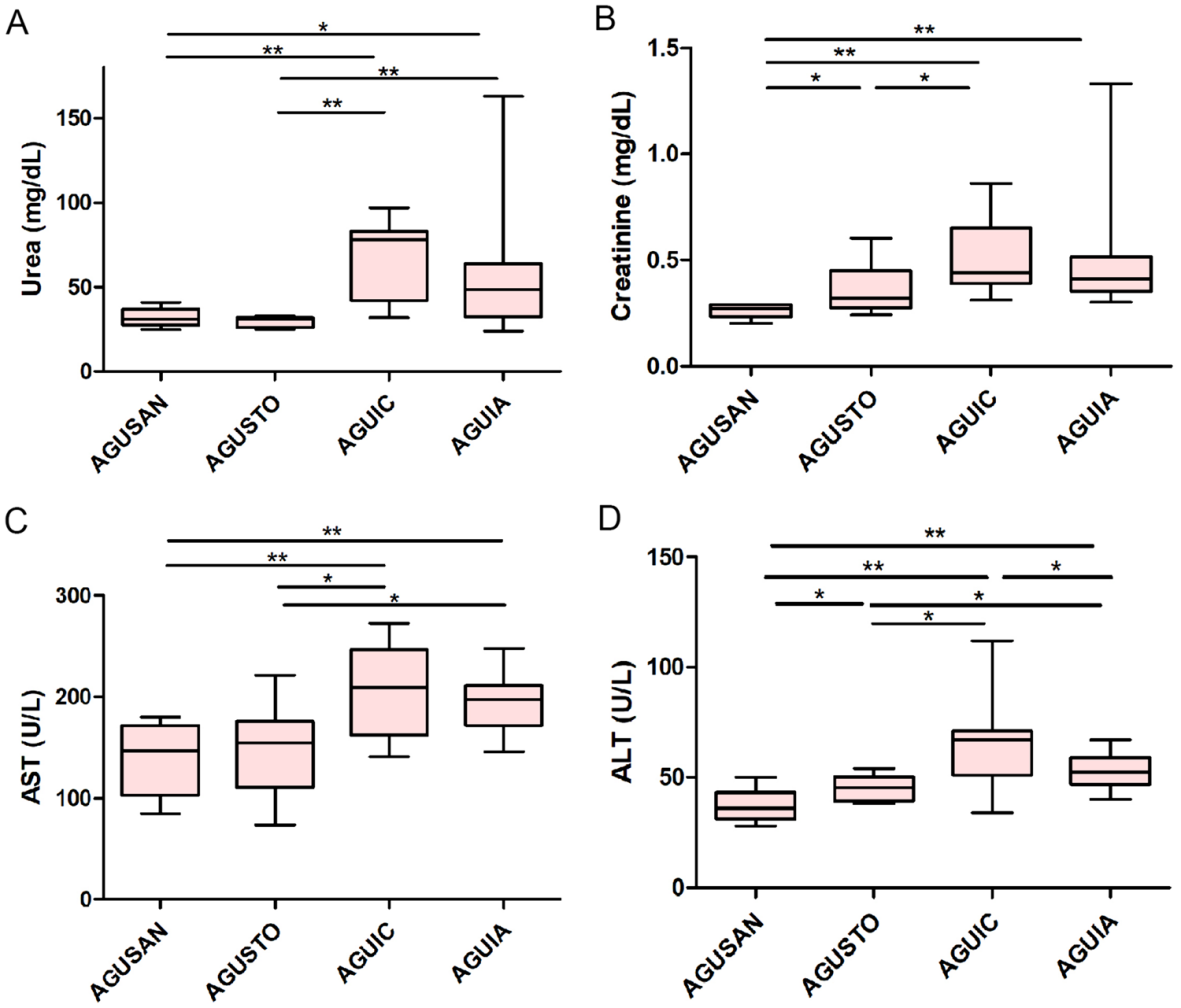
Serum levels of biochemical parameters (**A** urea, **B** creatinine, **C** aspartate aminotransferase (AST), and **D** alanine aminotransferase (ALT)) in healthy groups (AGUSAN and AGUSTO) and septic groups (AGUIC and AGUIA) at the time of euthanasia (phase 2). Box and whisker diagrams depict the smallest observation, lower quartile, median, upper quartile, and largest observation. *P < 0.05; **P < 0.01

Values of serum biochemical parameters are reported in Fig. 6. In general, AGUIC and AGUIA had significant increases (*P* < 0.05 and *P* < 0.01) in the levels of urea, creatinine, AST, and ALT when compared to AGUSAN and AGUSTO. Levels of creatinine (0.27 mg/dl vs. 0.32 mg/dl, *P* < 0.05) and ALT (36 mg/dl vs. 45.5 mg/dl, *P* < 0.05) were different between non-septic and septic groups.

### Microbiological Analysis in Blood, Feces, PLF, Urine, and BALF

**Fig. 7:**
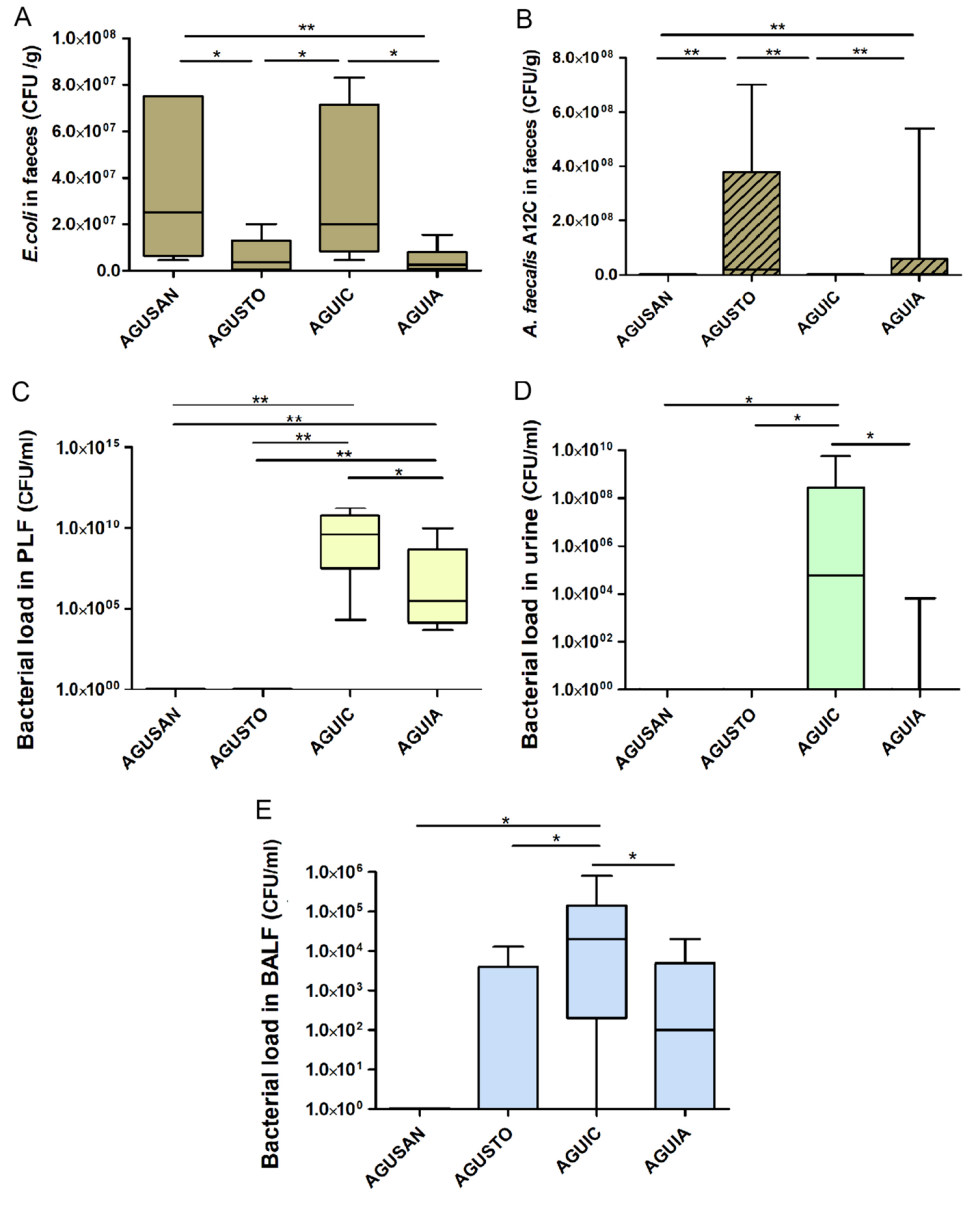
Feces concentration of *E. coli* (**A**) and *A. faecalis* A12C (**B**) at 30 days, after the first dose of probiotic in AGUSTO and AGUIA or only water, without probiotic, in AGUSAN and AGUIC. Bacterial load in PLF (**C**), urine (**D**), and BALF (**E**) at the time of euthanasia (phase 2). Box and whisker diagrams depict the smallest observation, lower quartile, median, upper quartile, and largest observation. *P < 0.05; **P < 0.01

All septic animals (AGUIC and AGUIA groups) had positive blood cultures; *E. coli* was the main isolated microorganism (Supplementary Table 3). All non-septic groups (AGUSAN and AGUSTO) had negative blood cultures. In feces, decreases of *E. coli* were observed in groups pretreated with *A. faecalis* A12C (AGUIA and AGUSTO) (*P* < 0.05 and *P* < 0.01, respectively) compared to the other two groups (Fig. 7A). *A. faecalis* A12C were found in higher amounts (*P* < 0.01) in groups pretreated for 30 days (AGUSTO and AGUIA) (2 × 10^7^ CFU/ml vs. 3 × 10^6^ CFU/ml, *P* < 0.01) (Fig. 7B). Aerobic bacterial load of PLF, urine, and BALF (Fig. 7C–E, respectively) decreased (*P* < 0.05) in AGUIA compared to AGUIC. In addition, the bacterial species were isolated from the different body fluids, corresponding to each animal, and their total percentages in each group (Supplementary Table 4), where *E. coli* were the most prevalence bacteria (92.9% vs. 73.3% in AGUIC vs. AGUIA group). Finally, the significant decrease in the number of urine samples with positive cultures in septic animals pretreated with *A. faecalis* A12C (AGUIA) was very striking compared to almost all septic animals without pretreatment (AGUIC), where *E. coli* returned to being the most frequently isolated bacterial species (90.9%).

### Cytokines and CRP Assays

**Fig. 8:**
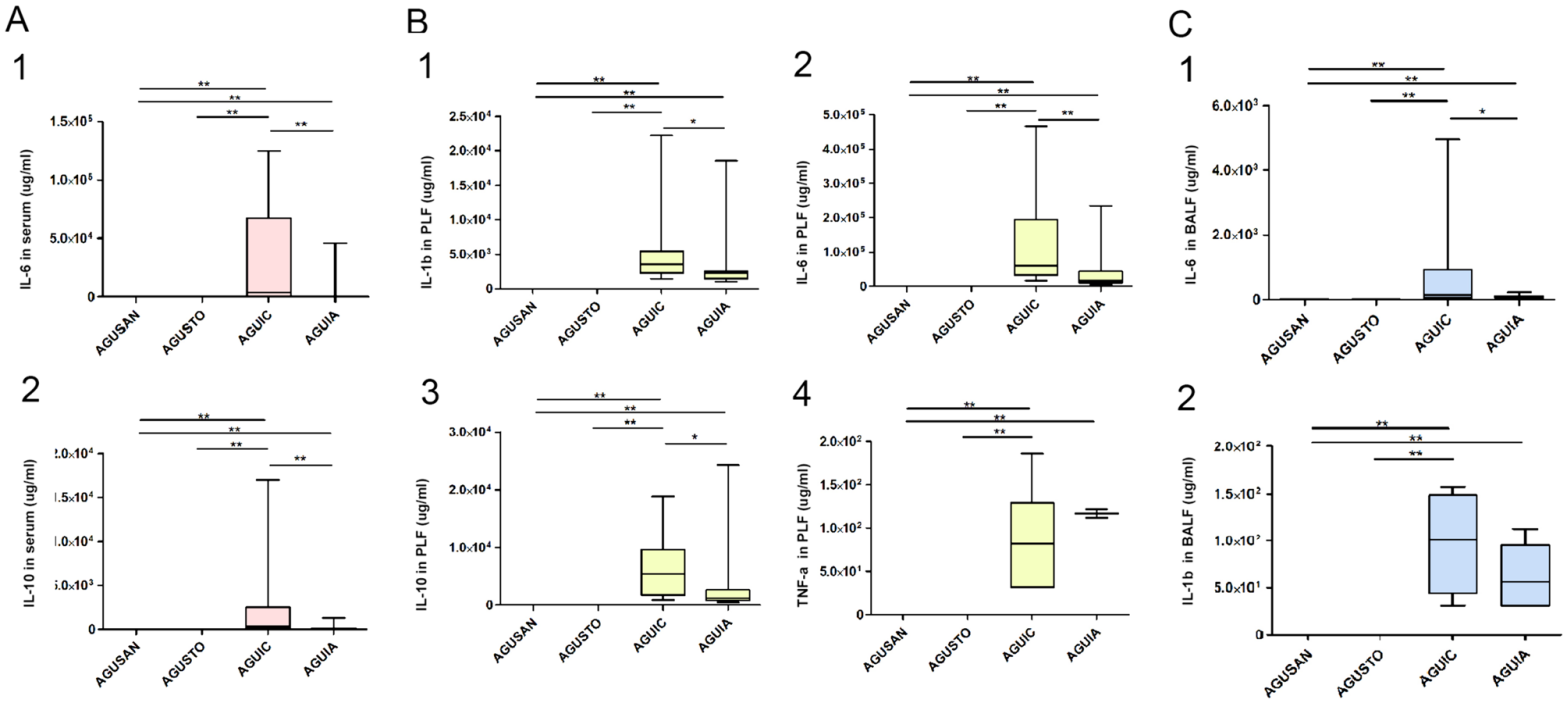
Effect of *A. faecalis* A12C pretreatment on inflammatory cytokines (IL-1β, IL-6, IL-10, and TNF-α) in AGUSTO and AGUIA and in their corresponding non-pretreated control groups (AGUSAN and AGUIC). Levels of cytokines in serum (**A**), PLF (**B**), and BALF (**C**). Box and whisker diagrams depict the smallest observation, lower quartile, median, upper quartile, and largest observation. *P < 0.05; **P < 0.01

**Fig. 9:**
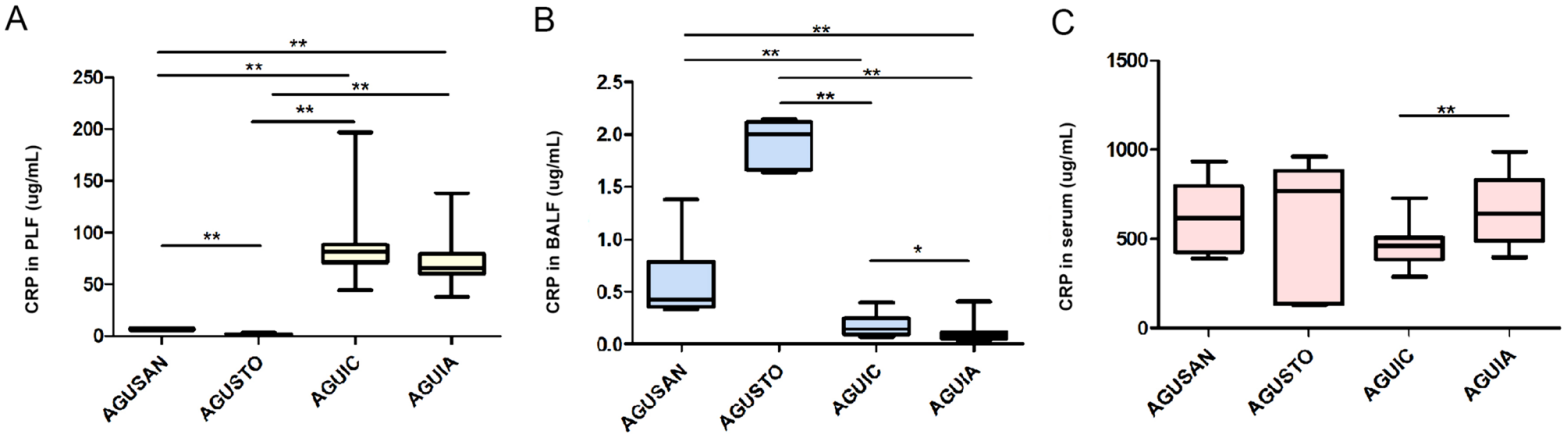
Effect of *A. faecalis* A12C pretreatment on C-reactive protein (CRP) in AGUSTO and AGUIA and in their corresponding non-pretreated control groups (AGUSAN and AGUIC). CRP levels in PLF (**A**), BALF (**B**), and serum (**C**). Box and whisker diagrams depict the smallest observation, lower quartile, median, upper quartile, and largest observation. *P < 0.05; **P < 0.01

IL-6 in serum (Fig. 8A (1)), PLF (Fig. 8B (1)), and BALF (Fig. 8C (1)) were all distinctly elevated after CLP (*P* < 0.01, *P* < 0.01, and *P* < 0.05, respectively) and mitigated by pretreatment with *A. faecalis* A12C. Similar results were observed in IL-10 in serum (Fig. 8A (2)) and PLF (Fig. 8B (2)). IL-1β increased in septic groups and attenuated in AGUIA group compared to AGUIC (Fig. 8B (3), C (2)) (*P* < 0.05). TNF-α was not detectable in serum nor in BALF; however, TNF increased in PLFs from AGUIC and AGUIA groups (Fig. 8B (4)). After 20 h of CLP, CRP concentration increased (*P* < 0.01) in PLF in AGUIC and AGUIA groups (Fig. 9A) and decreased in BALF in septic groups (Fig. 9B). The decrease in CRP was more pronounced in AGUIA than in AGUIC (*P* < 0.05). Serum CRP increased in AGUIA when compared to AGUIC (Fig. 9C) (*P* < 0.01).

### Curdlan Measurement

**Fig. 10:**
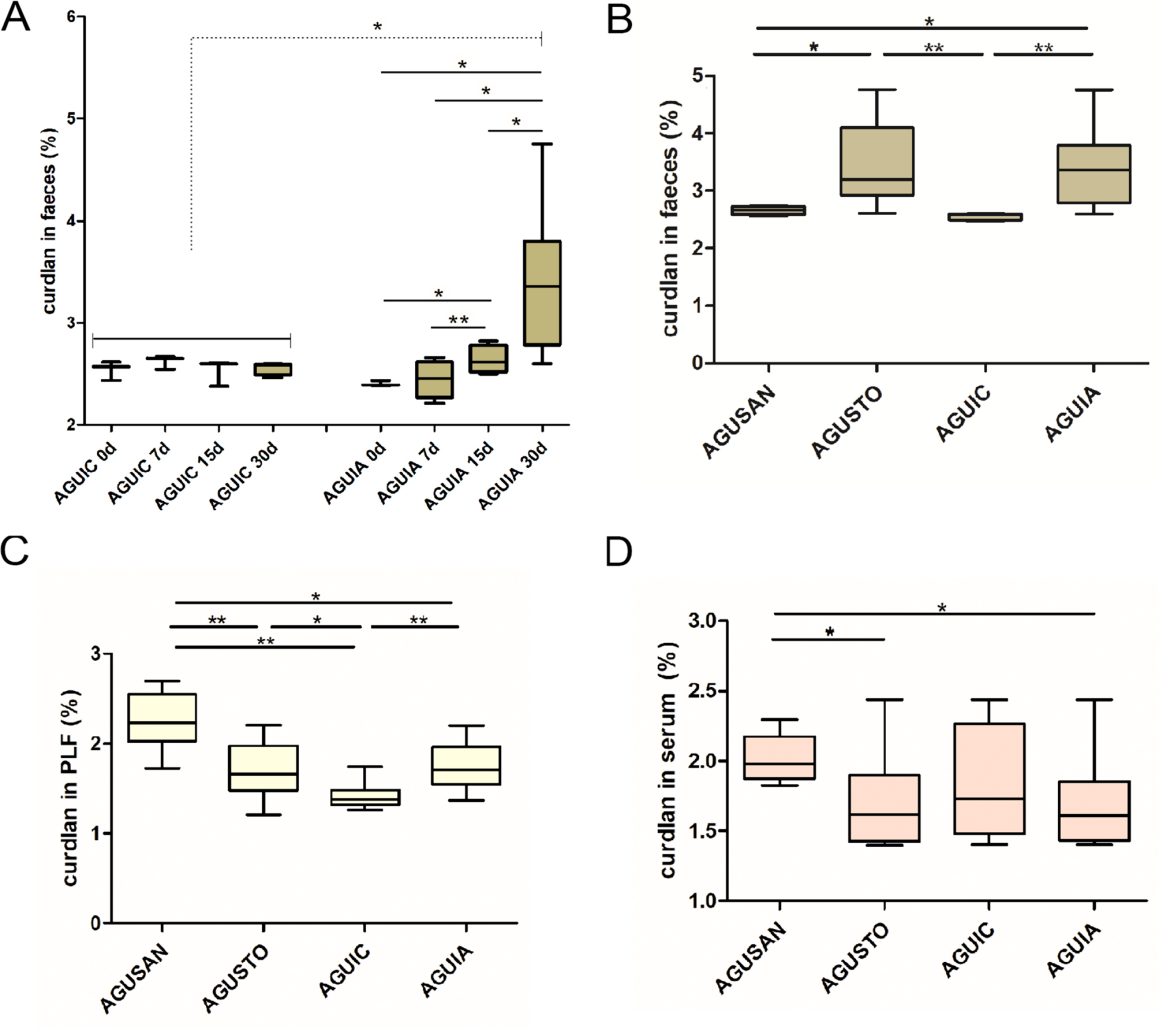
Feces concentrations of curdlan at different times in septic groups (AGUIC and AGUIA) (**A**) assessed at 0 day (0d; just before administration of *A. faecalis* A12C or only water), 7 days (7d), 15 days (15d), and 30 days (30d), after the first dose of probiotic or only water. Percentage of curdlan in feces (**B**) assessed at 30 days, after the first dose of probiotic in AGUSTO and AGUIA or only water, without probiotic, in AGUSAN and AGUIC (phase 2). Percentage of curdlan in PLF (**C**) and serum (**D**) in healthy groups (AGUSAN and AGUSTO) and septic groups (AGUIC and AGUIA) at the time of euthanasia (phase 2). Box and whisker diagrams depict the smallest observation, lower quartile, median, upper quartile, and largest observation. *P < 0.05; **P < 0.01

A progressive increase (*P* < 0.05) in the concentration of curdlan in feces in AGUIA group was found. In contrast, this beta-glucan remained stable throughout the 30 days in the AGUIC group (Fig. 10A). At 30 days after the administration of *A. faecalis* A12C, curdlan in feces increased in AGUSTO and AGUIA groups (*P* < 0.01) (Fig. 10B). Curdlan in PLF, in the rats of the AGUIA group compared to their AGUIC control group, showed a very significant increase (*P* < 0.01) similar to that previously described in feces. However, a very significant decrease (*P* < 0.01) was observed in the AGUSTO group compared to AGUSAN (Fig. 10C). Serum curdlan did not experience significant differences between AGUIC and AGUIA septic groups. On the other hand, a significant decrease (*P* < 0.05) in the amount of serum curdlan was observed in the AGUSTO group, compared to its control AGUSAN (Fig. 10D). Curdlan was not detectable neither in urine nor in BALF in any of the groups after administration of *A. faecalis* A12C (AGUSTO and AGUIA) or just water (AGUSAN and AGUIC).

### Cytological Evaluation of PLF, Urine, and BALF

**Fig. 11:**
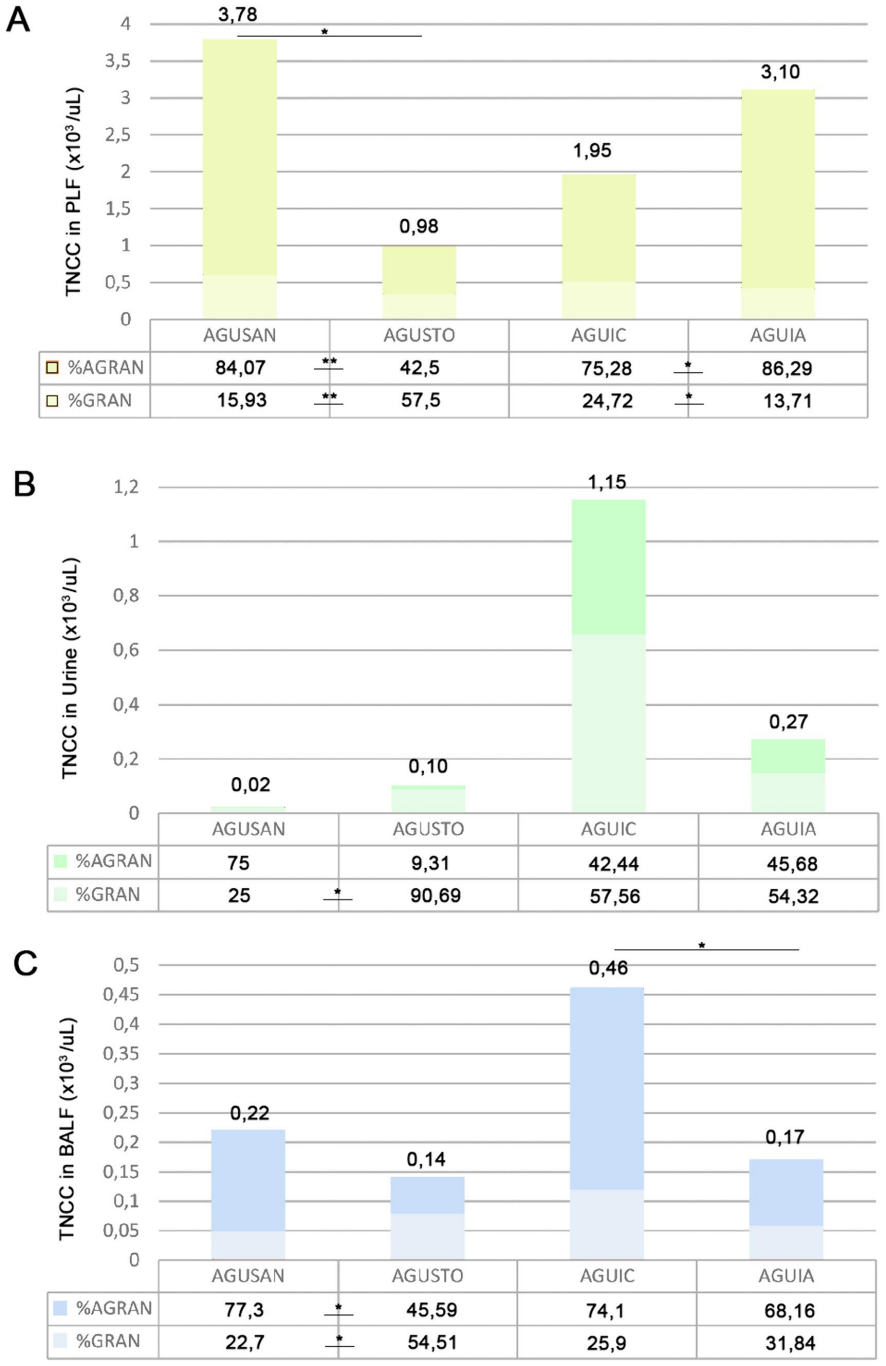
Effect of *A. faecalis* A12C pretreatment on total nucleated cell count (TNCC) and their corresponding percentages of agranulocytes (%AGRAN) and granulocytes (%GRAN) in PLF (**A**), urine (**B**), and BALF (**C**), in AGUSTO and AGUIA and in their corresponding non-pretreated control groups (AGUSAN and AGUIC). The results are expressed as the median (P50) from rats. *P < 0.05; **P < 0.01

In PLF (Fig. 11A), the results showed a trend towards an increase in granulocytes in AGUIC group compared to AGUIA (*P* < 0.05). On the other hand, there was a decrease in granulocytes in animals of AGUIC group with respect to AGUIA (*P* < 0.05). In urine (Fig. 11B), significant differences could be observed among groups, with a lower increase in the total number of nucleated cells in the AGUIA group (0.27 × 10^3^ cell/ml) than in the AGUIC group (1.15 × 10^3^ cell/ml). No differences were found between groups in the percentages of granulocytes and agranulocytes. In BALF (Fig. 11C), a similar trend was observed in the total number of nucleated cells, with a lower increase in the AGUIA group (0.17 × 10^3^ cell/ml) than in the AGUIC group (0.46 × 10^3^ cell/ml). In addition, a significant decrease in the percentage of granulocytes was also observed in AGUIC compared to AGUSAN (*P* < 0.05) and an increase in the percentage of agranulocytes between the same groups (*P* < 0.05). However, no differences were found either in the percentage of granulocytes or in the percentage of agranulocytes between the AGUSAN and AGUIA groups.

### Histological Evaluation and W/D Lung Weight Ratio

**Fig. 12:**
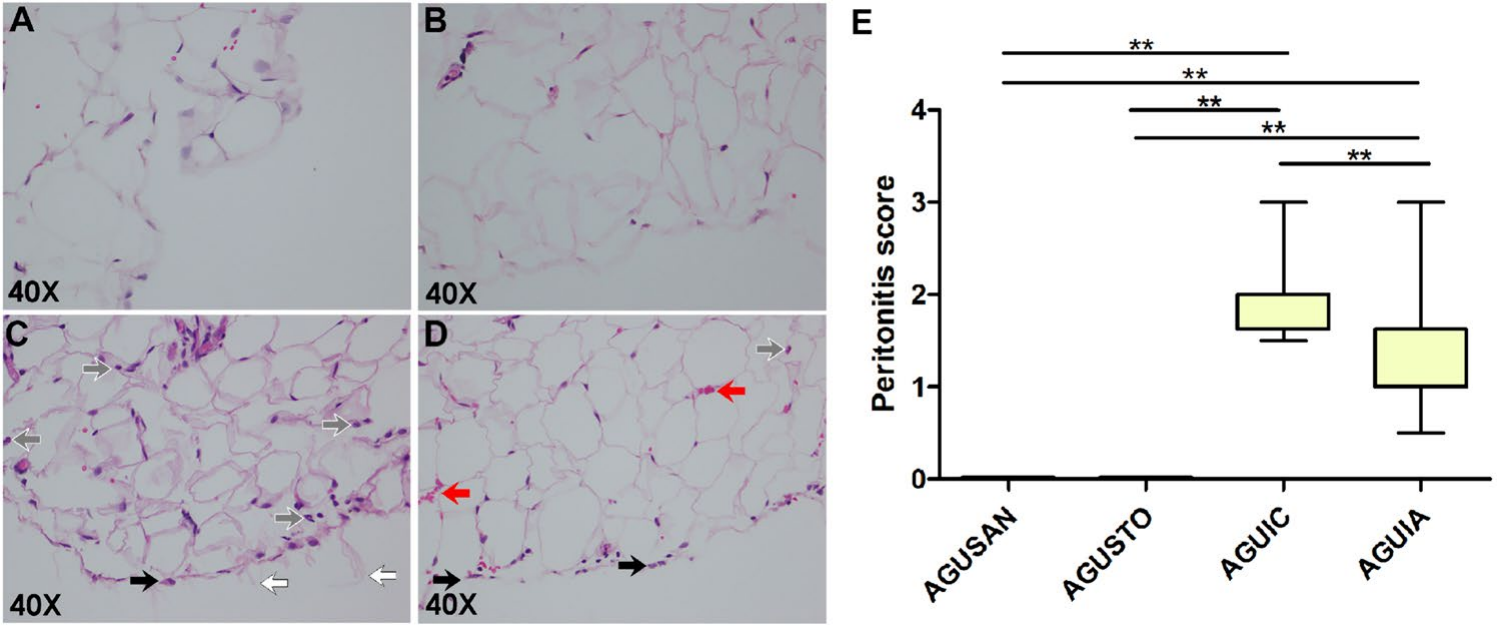
Representative histological images of hematoxylin and eosin–stained sections of jejunal peritoneum. No sign of inflammation or tissue alteration in peritoneal surface in a rat from AGUSAN group (**A**) neither in the other one belonging to AGUSTO group (**B**). Dulling of the peritoneal surface, swelling of mesothelial cell (black arrow), and leukocytic infiltrate (gray arrow) in jejunal peritoneum in a rat from AGUIC group (**C**) and, with less evidence, in the other one from AGUIA group (**D**). Focal desquamation of mesothelial cells (white arrow) in peritoneum from AGUIC group (**C**). Extravascular erythrocytes (red arrow) in a rat belonging to AGUIA group (**D**). **E** Severity of lung damage. **P < 0.01

**Fig. 13:**
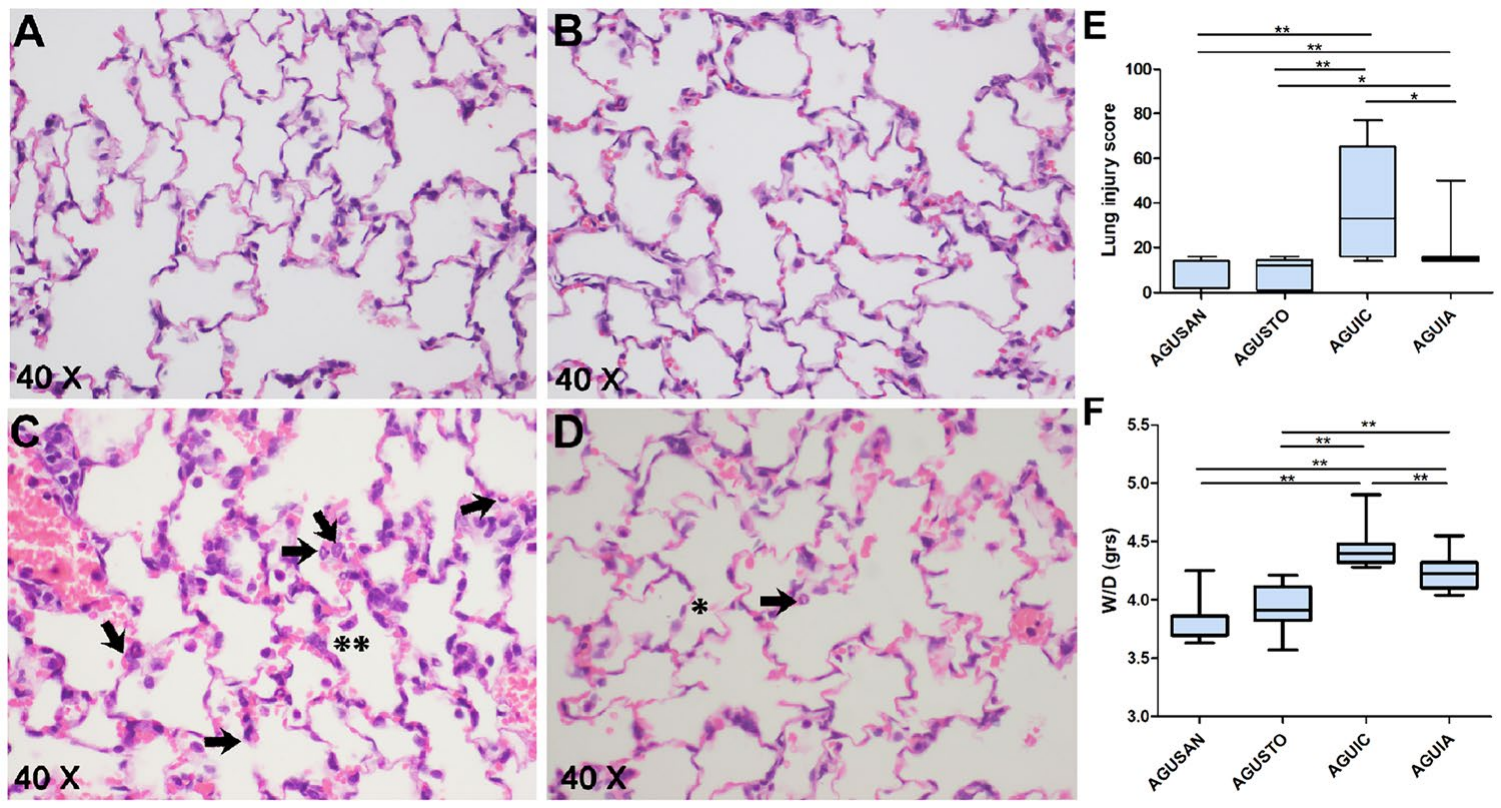
Representative histological images of hematoxylin and eosin–stained sections of lung. No sign of inflammation or tissue alteration in a rat from AGUSAN group (**A**) neither in the other one belonging to AGUSTO group (**B**). Neutrophils in the interstitial space (black arrow) in a rat from AGUIC group (**C**) and, with less evidence, in the other one from AGUIA group (**D**). Two to four times alveolar septal thickening (**) in a rat from AGUIC group (**C**) and less than two times alveolar septal thickening (*) in the other one from AGUIA group (**D**). *P < 0.05; **P < 0.01

Both septic groups had lesions compatible with peritonitis. Severity of lung damage was significantly lower in AGUIA than in AGUIC (*P* < 0.01) (Fig. 12E). Histologically, a similar situation could be observed in the case of lung lesions. Thus, there was less lung damage in AGUIA than in AGUIC (Fig. 13E), according to the lung injury score used (median 15 vs. 33, *P* < 0.05). Degree of pulmonary edema was analyzed based on the W/D lung weight ratio (Fig. 13F), and it was lower in AGUIA than in AGUIC (*P* < 0.01). No signs of peritoneal or pulmonary injury were found in the AGUSAN and AGUSTO groups. Except for peritoneum and lung, no significant histological changes were observed in any of other organs in the four groups. Representative histological lesions are shown in Figs. 12 and Fig. 13.

## Discussion

The primary discoveries of our research indicate the safety of *A. faecalis* A12C, which was isolated from *Argyrosomus regius*. It also suggests a potential link between the β-1,3-glucan (curdlan) produced by this specific strain and its impact on the immune system, resulting in a reduction in the severity of septic conditions. This study represents the initial exploration of the probiotic properties of *A. faecalis* and the influence of β-glucan produced by *A. faecalis* A12C as an immune response modulator in the widely recognized animal model of sepsis.

Previous in vitro and in vivo studies carried out with *A. faecalis* A12C demonstrated its probiotic crossed effect against vibriosis or *Photobacterium damselae* subsp. *piscicida* in relevant fish species for marine aquaculture [11, 32]. Recently, our research group has shown the potential probiotic effect of this strain in mammals. Administration of *A. faecalis* A12C in drinking water (6 × 10^8^ CFU/ml) for 7 days was associated in this study with a lesser spread of infection, obtaining promising clinical, microbiological, biochemical, and histopathological results in a rat model of peritonitis produced by *E. coli* [12]. These findings, together with the scarcity of studies focused on the probiotic properties of this strain [33], set us the objective of evaluating whether the administration of *A. faecalis* A12C would generate a preventive effect against the damage caused by sepsis, as well as to identify the possible immunomodulatory mechanisms of action in an experimental model in rats.

Thus, we analyzed the survival of *A. faecalis* A12C–pretreated septic animals. It was demonstrated that this strain improved the survival of septic rats. For sepsis induction, we have used the most common model described, cecal puncture ligation where survival rate, 24 h after CLP, is close to 80% in rats without any type of treatment, except analgesia and subcutaneous hydration [23]. However, in animals pretreated with *A. faecalis* A12C, the survival rate improved and was higher than expected for this technique at 24 h and were similar to that found by other authors at 48 h and 72oursh, in which they tested the activity of mixtures of probiotics included *Bifidobacterium longum*, *Lactobacillus bulgaricus*, and *Streptococcus thermophilus* in Wistar septic rats by CLP [2, 34]. In addition, the survival rate of healthy animals that were administered the strain was not affected. Regarding the clinical signs due to sepsis and although there are authors who find clinical signs similar to ours [12, 2], there are no papers with quantitative studies of these variables using the rat CLP model in probiotic efficacy studies.

Feces and fluid microbiological study showed a significant decrease in the fecal concentration of *E. coli* in healthy and septic groups pretreated with *A. faecalis* A12C (AGUIC and AGUIA). This finding has already been described by our group in a peritonitis model [12], suggesting a competitive effect of *A. faecalis* A12C on *E. coli*. Other authors have shown that the use of *Lactobacillus* spp. in broilers, orally, also reduces the concentration of *E. coli* in the intestine [35]. In addition, pigs fed for 2 weeks after weaning with β-glucans from *Saccharomyces cerevisiae* and/or *Sclerotium rolfsii* were less susceptible to an F4 antigen + enterotoxigenic *E. coli* infection in comparison with the control group. This was evidenced by a reduction in the fecal excretion of F4 + *E. coli* as well as a reduced F4-specific serum antibody response [18]. CLP is considered as the gold standard model for studying sepsis in animals which closely mimics human sepsis and isolating *E. coli* very frequently in various biological samples [23, 36]. However, microbial identification is not a common practice in preclinical models of sepsis [37]. In this way, one of the most interesting results of this study has been to verify a decrease in the number of samples which *E. coli* was isolated and the greater variety of bacterial species isolated in the PLF and BALF samples in the pretreated septic group (AGUIA) compared to septic animals without pretreatment (AGUIC). In a study based on bacterial identification in blood and PLF samples in Wistar rat’s septic by CLP, with study times similar to ours, they have reported that *E. coli* (88%), *Enterococcus faecalis* (81%), and *Enterobacter cloacae* (75%) were the main pathogens found in blood cultures. However, *E. coli* (75%), *Enterococcus faecalis* (94%), and *Lactobacillus murinus* (69%) were the main pathogens found in PLF cultures [37]. These results are in partial agreement with ours since *E. coli* was also the most frequent species in the blood cultures; however, *Lactobacillus murinus* was isolated also, being more frequent in the AGUIA blood cultures than in AGUIC. In the bacteria isolated from PLF, we also agree with these authors, since the species mostly isolated were *E. coli*, *Enterococcus faecalis*, and *Lactobacillus murinus*, although the percentages of isolates are lower. Analyzing the results of the BALF samples, it was striking to observe that none of the species that we isolated and identified in healthy animals with (AGUSTO) or without (AGUSAN) pretreatment with *A. faecalis* A12C (*Muribacterium muris*, *Klebsiella pneumoniae*, *Corynebacterium striatum*, *Lactobacillus paracasei*, and *Staphylococcus* spp. (coag -)) was isolated in the groups of septic animals (AGUIC and AGUIA), where *E. coli*, *Rodentibacter pneumotropicus*, *Staphylococcus sciuri*, and *Haemophilus haemolyticus* were the most frequently isolated species. In summary, our results are in accordance with common polymicrobial infections in stercoral peritonitis in humans, and similar microbial profile (*Enterobacteriaceae* and *Enterococci*) was also found between our CLP model and human peritonitis [37, 38].We think that the microbiological diagnostic methodology used in this work, focused on an effort for the intentional search for both aerobic and anaerobic bacterial species and a powerful and highly reliable identification system such as MALDI-TOF, has been decisive in obtaining a more accurate information on the spread of infection by the different biological fluids.

It is well known that the main molecules of β-glucans are found in the cell wall of a wide range of bacteria, fungi, yeasts, plants, and algae. In our study, we observed a significant increase in the fecal concentrations of curdlan in pretreated groups with *A. faecalis* A12C compared to the untreated groups. This could be explained by the fact that some authors have reported that certain β-glucans can pass undigested through the gastrointestinal tract, acting as a substrate for microbial fermentation and selectively stimulating the growth and activity of a small number of bacteria [39, 40]. In PLF, a significant increase in curdlan concentration was observed in the groups pretreated with *A. faecalis* A12C compared to the AGUIC group. These findings are consistent with results reported by other researchers in pilot studies, where β-glucans are used as biomarkers for the diagnosis of fungal peritonitis, detecting concentrations of β-glucans of both bacterial and fungal origins [41]. This aligns with our previous findings [12], which revealed an increase in the quantity of *A. faecalis* A12C in our study, which contains curdlan. Furthermore, the detection of curdlan in PLF could be attributed to the transmural migration of gastrointestinal macromolecules produced during intestinal inflammation, as indicated by other previous studies [42–44]. Regarding curdlan levels in serum, we found a small but significant decrease in the pretreated groups (AGUSTO and AGUIA) compared to the healthy rat group (AGUSAN). Other authors have reported that they did not detect any changes in orally administered β-glucan levels during their 14-day study [45]. This may suggest that the decrease we observed in our study could be attributed to the curdlan’s mechanism of action with certain immune cells present in the blood, such as monocytes or macrophages favoring its phagocytosis and transfer to other organs such as the spleen [16]. Urine and BALF curdlan concentrations were unable to detect, and this may be due to the fact that, during the lyophilization process, the minimum required sample quantity for β-glucan detection was not met, highlighting an area for improvement in future studies.

**Fig. 14:**
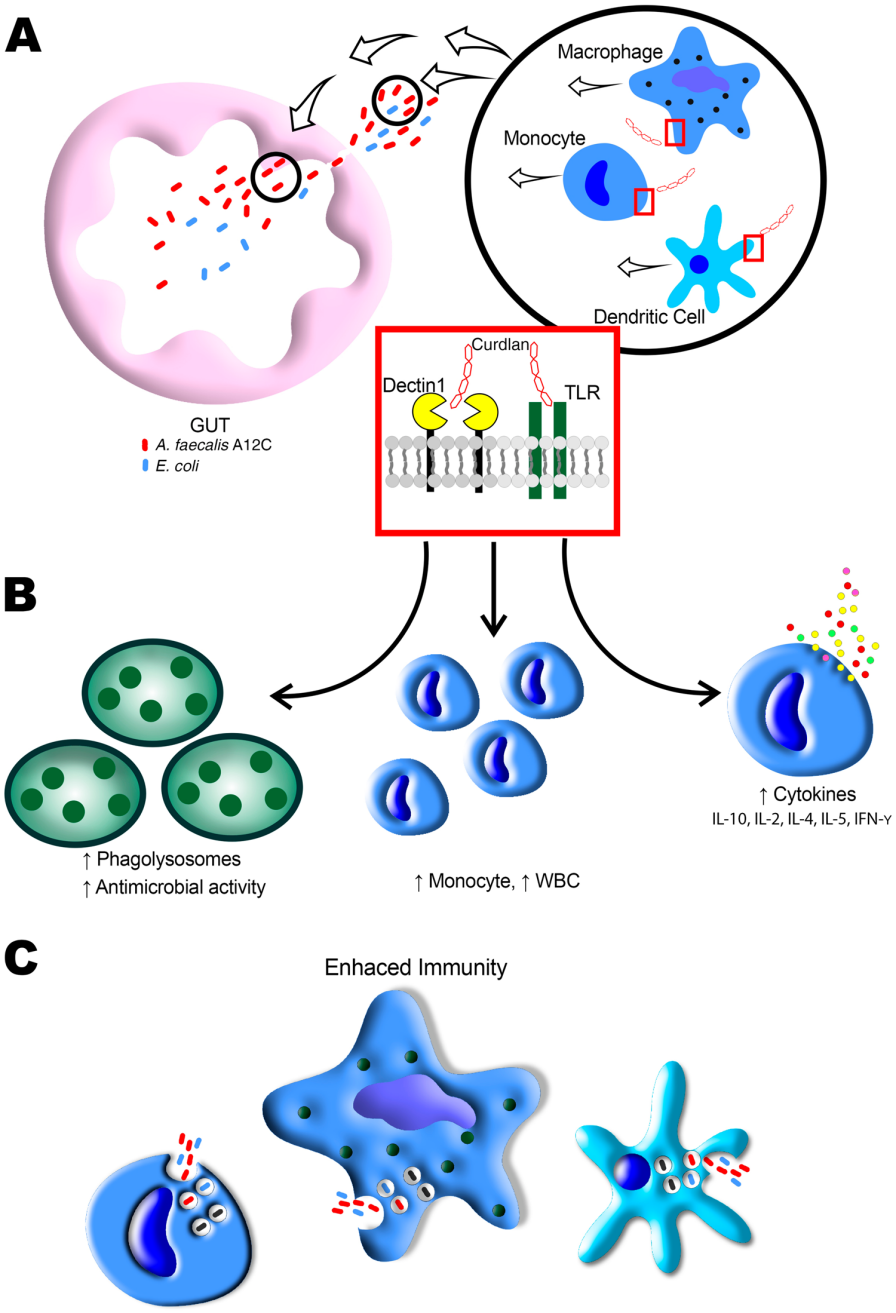
Graphic representation of the process occurring after the administration of *A. faecalis* A12C, adapted from the referenced studies [16, 50, 52]. A higher bacterial load of *A. faecalis* A12C is observed in the intestine compared to *E. coli* (**A**). Following gut rupture and subsequent peritonitis, macrophages, monocytes, and dendritic cells are recruited to the affected area. These cells recognize and bind to the β-1,3/1,6-glucan chains, specifically curdlan, through dectin-1 receptor and Toll-like receptor (TLR). This interaction triggers an increase in phagolysosome formation, production of reactive oxygen species through Syk and Src kinases, and improved antimicrobial activity, as well as an elevation in the concentration of monocytes and white blood cells (WBC). Dectin-1 collaborates with other receptors such as TLRs in the induction of pro-inflammatory cytokine production to activate NF-κB (**B**). Finally, an enhanced immunity response occurs, in which the primary immune cells phagocytize the pathogens present in the environment (**C**)

Some of the most important interleukins in septic process were measured in serum, BALF, and PLF by ELISA (IL-6, IL-1β, TNF-α, IL-10). Non-septic animals presented undetectable levels of cytokines (AGUSAN, AGUSTO). However, in septic animals, an increase in IL-6, IL-1β, and IL-10 levels in non-pretreated group (AGUIC) vs. pretreated group (AGUIA) was observed, but no significant changes were observed for TNF-α in PLF. These results showed a decrease of some pro-inflammatory and anti-inflammatory cytokines, and similar results were described in a lung injury rodent model by CLP treated with probiotics *Lactobacillus rhamnosus* GG and *Bifidobacterium longum* [46] or in a recent study about the evaluation of *Lactobacillus plantarum* and *Lactobacillus acidophilus* on the efficacy of immune system in male and female Wistar rats [47]. In regard to these results, we propose that immunomodulation may play an important role in how the immune system acts against pathogen. Many experimental and clinical studies have shown that sepsis induces multiple organ dysfunction syndrome (MODS), where the lungs are ones of the most important organ affected and often it leads to acute respiratory distress syndrome that is caused by an uncontrolled and complex interaction between inflammatory cytokines and cellular mediators that produce alveolocapillary damage [31, 36, 48]. In this way, it has been proposed that early attenuation of these pro-inflammatory cytokines is a goal in sepsis treatment [49]. Immunomodulatory effect of *A. faecalis* A12C can lead to an improvement in MODS through downregulating inflammatory cytokines and cellular mediators, making a smarter response against infection, where curdlan may play an important role. There are some authors that describe how β-glucans improve innate immune response–enhanced antimicrobial and inflammatory properties derived from dectin-1/Toll-like receptor (TLR) activation (Fig. 14) [16]. It has been described that daily consumption of β-glucans was associated with fewer episodes and shorter duration of acute respiratory infections in children, as well as less antibiotic use. The children who consumed β-glucans had increased serum IL-10 and white blood cells, suggesting an anti-inflammatory mechanism and/or an increase in effector immune cells [50]. Some clinical trials evaluating the in vivo effects of orally administered β-glucans on blood effects have suggested an increased activation of circulating leucocyte and monocyte concentrations, altered monocyte cell-surface receptors, increased T cell concentration, increased serum IFN-γ, and an increase in LPS-stimulated production of IFN-γ and IL-2 [50–52]. These findings are consistent, partially, with the significant elevation in the total number of white blood cells and blood monocyte concentrations that we have found, both in healthy pretreated animals (AGUSTO) and in septic pretreated animals (AGUIA) compared to their respective controls without *A. faecalis* A12C (AGUSAN and AGUIC). This could be explained by the fact that β-glucans are phagocytosed and processed by monocytes and macrophages found in the intestinal lymphatic tissue, and then transported to different immune organs to prepare immune cells for an antimicrobial and inflammatory response against potential pathogens [16]. In contrast, in other in vitro studies where β-glucans from *A. faecalis* are administrated in not pathologic conditions, these produce an increase in the expression of M1 macrophages that have a pro-inflammatory phenotype with pathogen-killing abilities, upregulating IL-6, IL-1β, TNF-α, and MCP-1 expression [53, 54]. As in our case, in which we have not detected differences in the concentrations of cytokines in the different fluids analyzed between the healthy animals (AGUSAN) and the healthy ones pretreated (AGUSTO), in a study of ex vivo stimulation of leukocytes from healthy adults who received β-glucans, it was observed that no β-glucans were detected in serum. In addition, leukocyte cytokines were not altered compared to controls [45].

Regarding biochemical markers of liver and kidney damage, we have observed that there is a slight elevation of creatinine and ALT in the AGUSTO group compared to AGUSAN. This increase in serum creatinine has also been observed in healthy Wistar female rats treated with *Lactobacillus* spp. [47], and as in our case, there are authors who have observed a non-significant increase in ALT and AST in rats of both sexes that were given a strain of *Lactobacillus paracasei* [55]. However, the significant decrease in the concentration of ALT, which we have observed in septic animals pretreated (AGUIA) compared to septic animals without pretreatment (AGUIC), could suggest that *A. faecalis* A12C has a certain hepatoprotective effect during sepsis. Many studies have investigated the possible effect of probiotics on the concentrations of liver enzymes. Most of the research has concluded that administration of different probiotics strains to experimental animals not only induces the increase of serum liver enzymes, but also reduces the amounts of these enzymes [55–58].

The lungs are one of the first organs primed by local and/or systemic cytokine storm during sepsis, and they are damaged both morphologically and functionally. There is increasing acknowledgment that the cellular makeup of the inflammatory response may not be limited to neutrophils alone. Instead, it varies depending on the specific model system employed and the stage of injury. Consequently, recent revisions in the assessment of experimental acute lung injury (ALI) in animal studies suggest the consideration of elevated levels of inflammatory monocyte and macrophage (and/or lymphocyte) subgroups in BALF or lung tissue as a significant characteristic. This likely reflects the importance of these infiltrating cell types in scenarios such as lung injuries induced by bacteria or viruses, as well as in cases of sterile injury [59]. Based on this and following the recommendations of the American Thoracic Society for the evaluation of lung damage in animal models, we have chosen to add, to the histopathological measurement of lung damage, the W/D index for measuring changes in lung vascular and epithelial permeability, and the cell concentration in BALF for measuring inflammation. In this way, we were able to observe an excellent correlation between the W/D index, the histological damage score, and BALF cellular concentrations (TNCC). The marked statistically significant decrease in the three variables studied in the AGUIA group compared to the AGUIC group suggests an important protective effect on the lung of the *A. faecalis* A12C strain. Similar effects on lungs have been described by other authors, in both clinical and experimental studies, when the therapeutic potential of some strains as probiotics (*Lactobacillus paracasei*, *Saccharomyces boulardii*, *Bifidobacterium*, *Lactobacillus*, *Enterococcus*, and *Bacillus*) was assessed [60–62].

At the peritoneal level, it was possible to observe an evident anti-inflammatory effect in AGUIA group compared to that in AGUIC. Histopathologically, a decrease in the score used was observed, due to less desquamation of mesothelial cells, less degree of infiltration of inflammatory cells, as well as less thickening of the peritoneal surface. These results are in accordance with those recently obtained by our group, in a model of fecal peritonitis due to *E. coli* inoculation where the protective role of *A. faecalis* A12C was evaluated [12]. In addition, the significant increase, in the AGUIA group, in the concentration of agranulocytes in PLF could be associated with the effect of proliferation and activation of macrophages and monocytes that *A. faecalis* A12C, through curdlan, could be produced in the focus of the infection as previously described by other authors [16]. Recent studies have demonstrated this proliferation of monocytes and macrophages in the peritoneum of rats after administration of *Lactobacillus rhamnosus* [63]. In regard to histopathological evaluation in liver, spleen, kidney, thymus, and heart, no injury signs were found. Nevertheless, some authors describe inflammatory infiltrate, cell wall disorder, interstitial edema, and interstitial area hemorrhage among other tissue injuries in similar models [64–66]. However, most studies report these damages 24 h after CLP when our evaluation is 20 h post-CLP, even though other studies showed early damage in mice before 20 h post-CLP [67, 68].

Our study exhibits some weaknesses and strong points. First, we do not know the possible late effects that *A. faecalis* A12C may cause in rats after 30 days of administration, as well as the safety and efficacy ranges at different doses. Second, the results of this work are obtained from research in male rats, and we do not know if the hormonal cycles in the female sex could interfere in the modulation of the immune response that *A. faecalis* A12C has shown in males. Third, although the enzymatic technique for the quantification of curdlan used in this work is recommended and validated, we think that in the future, we must modify it or use other more sensitive and specific ones for liquid samples, which allow us to detect this β-1,3-glucan in urine or BALF, where it was not detectable, possibly due to its low concentration. Fourth, in our study, we have focused on the microbiological differences between our animal groups for bacterial levels, without considering the essential role that virus and fungi could play in gut microenvironment.

However, the major strength of this study is that we used a potent and translational model of sepsis that allowed us to obtain results of clinical, hematological, biochemical, histopathological, immunological, and microbiological relevance with a global vision of the potential therapeutics used of *A. faecalis* A12C, and the possible immunomodulatory role that its metabolite, curdlan, could be playing.

In summary, our findings suggest that *A. faecalis* A12C could influence clinically relevant parameters in sepsis. Administration of 6 × 10^8^ CFU/ml water, orally for 30 days, was associated with a less spread of infection, a modification in cytokine concentrations and a survival increase. In this context, our study may add a piece to the complex puzzle of bacteria–host interactions and new therapeutic possibilities in the prophylaxis of sepsis. However, more studies are needed to allow us know in greater detail the immunomodulatory mechanisms of action of *A. faecalis* A12C and, specially, its metabolite curdlan.

## Supplementary Information

Below is the link to the electronic supplementary material.

Supplementary file1 (DOCX 107 KB)

Supplementary file2 (DOCX 21 KB)

Supplementary file3 (DOCX 18 KB)

Supplementary file4 (DOCX 16 KB)

Supplementary file5 (DOCX 17 KB)

Supplementary file6 (DOCX 23 KB)

Supplementary file7 (DOCX 23 KB)

## Data Availability

The datasets generated and/or analysed during the current study are available from the corresponding author on reasonable request.
