# Supplementary material for: Long-Term Treatment with Alcaligenes faecalis A12C Improves Host Resistance to Pathogens in Septic Rats: Possible Contribution of Curdlan-Like Immune Trainer
Source: Probiotics Antimicrob Proteins. 2024 Apr 26;17(5):3100–19. doi: 10.1007/s12602-024-10252-0 (PMC12532692; doi:10.1007/s12602-024-10252-0)
Supplement: Supplementary file 1 — Supplementary file1 (DOCX 107 KB) [file 12602_2024_10252_MOESM1_ESM.docx]

**SUPPLEMENTARY DATA**

**Supplementary Fig 1.** Body weight at different times in septic and healthy groups pretreated or no-pretreated with *A. faecalis* A12C.


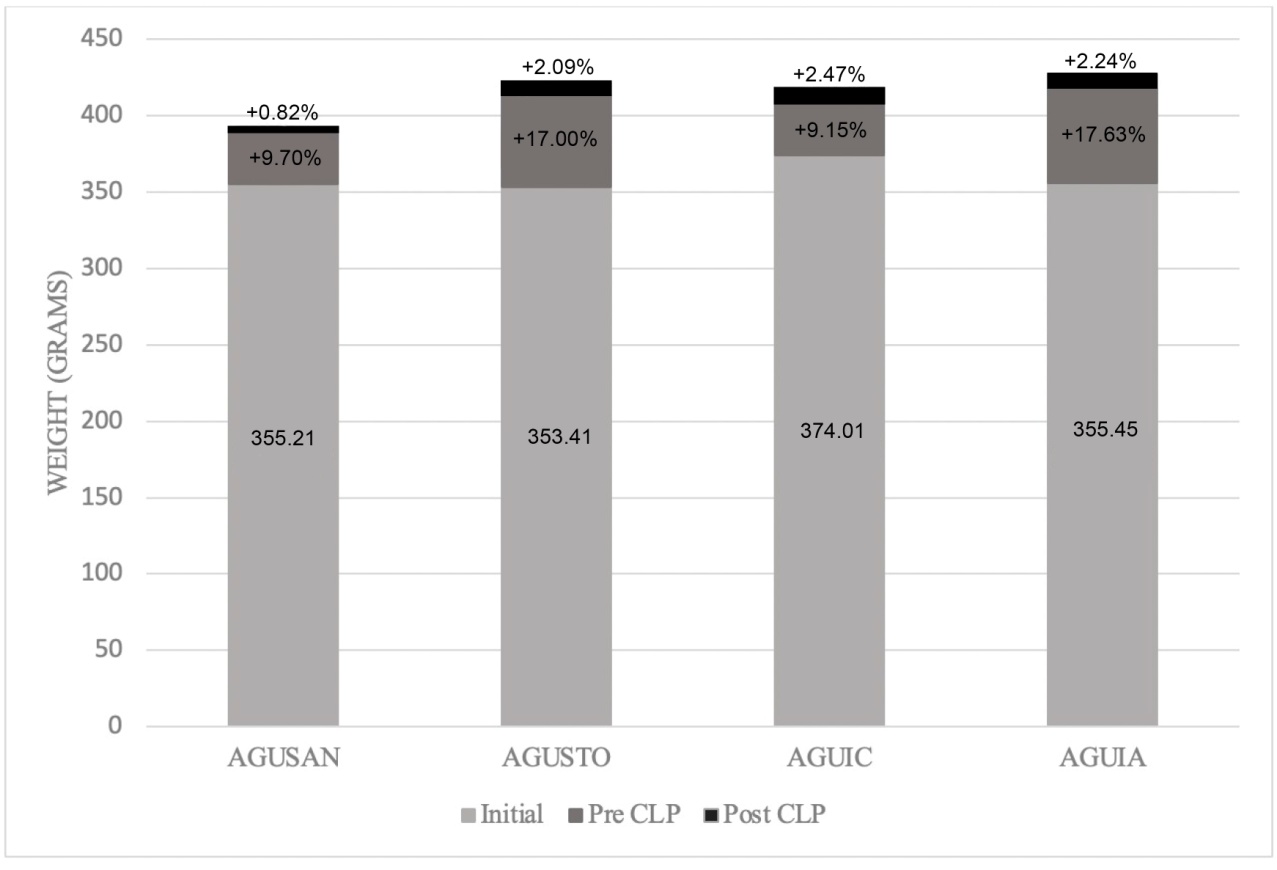


The results are expressed as the body weight mean in grams and % of weight gained, assessed at 0 days, 30 days, and 20h post CLP (AGUIC and AGUIA) or immediately before euthanasia (without CLP) in the case of healthy animals (AGUSAN and AGUSTO).
