# Supplementary material for: Long-Term Treatment with Alcaligenes faecalis A12C Improves Host Resistance to Pathogens in Septic Rats: Possible Contribution of Curdlan-Like Immune Trainer
Source: Probiotics Antimicrob Proteins. 2024 Apr 26;17(5):3100–19. doi: 10.1007/s12602-024-10252-0 (PMC12532692; doi:10.1007/s12602-024-10252-0)
Supplement: Supplementary file 2 — Supplementary file2 (DOCX 21 KB) [file 12602_2024_10252_MOESM2_ESM.docx]

SUPPLEMENTARY MATERIALS AND METHODS

**Long-term treatment with *Alcaligenes faecalis* A12C improves host resistance to pathogens in septic rats: possible contribution of curdlan-like immune-trainer**

**^#1^C.J. Martel-Benítez** (ORCID iD: 0000-0002-1580-4058)

**^#1^R. Alayón-Afonso** (ORCID iD: 0000-0002-2928-0144)

**^*1^D.F. Padilla Castillo** (ORCID iD: 0000-0002-6678-5029)

**^2^F.J. Chamizo López** (ORCID iD: 0000-0003-1328-1924)

**^3,4^M. Isabel García-Laorden** (ORCID iD: 0000-0001-6270-6306)

**^5^ª. Espinosa de los Monteros y Zayas** (ORCID iD: 0000-0002-7736-3139)

**^6^J.C. Rivero-Vera** (ORCID iD: 0000-0003-3039-0030)

**^7^P. Nogueira Salgueiro** (ORCID iD: 0000-0002-8029-2685)

**^1^F. Real Valcárcel** (ORCID iD: 0000-0001-6526-0354)

**^2^ª. Bordes Benítez** (ORCID iD: 0000-0003-3243-7402)

^8^**ª. Martel Quintana** (ORCID iD: 0000-0002-7450-0505)

^9^**C. Almeida Peña** (ORCID iD: 0000-0001-9283-4851)

**^7^C. Domínguez Cabrera** (ORCID iD: 0000-0003-2600-1637)

**^10^J.M. González-Martín** (ORCID iD: 0000-0001-6816-4157)

**^11^J. Martín Caballero** (ORCID iD: 0000-0002-1579-2739)

**^12^R. Frías Beneyto** (ORCID iD: 0000-0001-7569-5693)

**^3,4^Jesús Villar** (ORCID iD: 0000-0001-5687-3562)

**^1,13,14,15^J.L. Martín-Barrasa,** (ORCID iD: 0000-0002-3280-9838)

From

*(1) Fish Health and Infectious Diseases Group, University Institute of Animal Health and Food Safety (IUSA), University of Las Palmas de Gran Canaria, Carretera de Trasmontana s/n, 35416 Arucas, Spain;*

*(2) Microbiology Department. Hospital Universitario de Gran Canaria Dr Negrín, Barranco de la Ballena s/n, 35019 Las Palmas de Gran Canaria, Spain;*

*(3) CIBER de Enfermedades Respiratorias, Instituto de Salud Carlos III, Monforte de Lemos 3-5, Pabellón 11, 28029 Madrid, Spain;*

*(4) Multidisciplinary Organ Dysfunction Evaluation Research Network, Research Unit, Hospital Universitario de Gran Canaria Dr. Negrín, Barranco de la Ballena s/n, 35019 Las Palmas de Gran Canaria, Spain;*

*(5) Morphology Department. University Institute of Animal Health and Food Safety (IUSA). Universidad de Las Palmas de Gran Canaria. Arucas. Las Palmas. Spain;*

*(6) Pathology Service. Hospital Universitario de Gran Canaria Dr Negrín, Barranco de la Ballena s/n, 35019 Las Palmas de Gran Canaria, Spain;*

*(7) Clinical Biochemistry Department. Hospital Universitario de Gran Canaria Dr Negrín, Barranco de la Ballena s/n, 35019 Las Palmas de Gran Canaria, Spain;*

*(8) Banco Español de Algas, Instituto de Oceanografía y Cambio Global, Universidad de Las Palmas de Gran Canaria, Telde, Spain;*

*(9) Banco Español de Algas, Fundación Parque Científico Tecnológico, Universidad de Las Palmas de Gran Canaria, Telde, Spain;*

*(10) Statistics Service. Research Unit, Hospital Universitario de Gran Canaria Dr Negrín, Barranco de la Ballena s/n, 35019 Las Palmas de Gran Canaria, Spain;*

*(11) Barcelona Biomedical Research Park (PRBB), Barcelona, Spain;*

*(12) Comparative Medicine, Karolinska Institutet, Stockholm, Sweden;*

*(13) Animal Facility, Research Unit, Hospital Universitario de Gran Canaria Dr Negrín, Barranco de la Ballena s/n, 35019 Las Palmas de Gran Canaria, Spain;*

*(14) Fundación Canaria del Instituto de Investigación Sanitaria de Canarias (FIISC), Las Palmas de Gran Canaria Spain;*

*(15) CIBER de Enfermedades Infecciosas (CIBERINFEC), Instituto de Salud Carlos III, Madrid, Spain.*

*#* Contributed equally

**∗Corresponding author:** DF Padilla Castillo**.** *Animal Infectious Diseases and Ictiopathology, University Institute of Animal Health and Food Safety, Universidad de Las Palmas de Gran Canaria, Carretera de Trasmontaña s/n, 35416 Arucas, Spain.* Phone: +(34)928459741. E-mail: daniel.padilla@ulpgc.es

**Animals**

Before the study, all rats underwent a 21-day acclimatization period. Each rat's health was confirmed through individual physical examinations, and they were declared pathogen-free following routine microbiological screening in adherence to European recommendations [28].

**Preparation of A. faecalis A12C**

*A.faecalis* was suspended in drinking mineral water (Fonteide®), and its bacterial concentration was measured at 600 nm using a spectrometer. The suspension was adjusted with mineral water (Fonteide®) to achieve a concentration of 6 × 10^8^ CFU/mL. This suspension was stored for a maximum of 48 hours at 4 °C, shielded from light, and renewed every 48 hours.

**Sepsis model**

The CLP model was employed to closely replicate human sepsis through peritonitis. Animal welfare was monitored every 8 hours, considering the signs outlined in Supplementary Table 1 [30]. For post-operative pain management, buprenorphine (Buprex®, Indivior Europe Ltd, Dublin, Ireland) was administered at a dose of 0.05 mg/kg every 8 hours. If the clinical welfare indicators were not met, analgesics could be administered every 6 hours.

**Blood Count and Blood Chemistry**

Whole blood samples were drawn from the jugular vein and collected in EDTA K3 vacutainers. Analysis was conducted using an autoanalyzer IDEXX ProCyte DxTM (IDEXX Laboratories Inc., Westbrook, Maine, USA) with settings tailored for rats.

Biochemical parameters were assessed using whole blood obtained via cardiac puncture and analyzed with the Cobas 8000 Modular Analyser Series (Roche Diagnostics, Basel, Switzerland).

Serum levels of C-reactive protein (CRP) were determined using an ELISA kit in accordance with the manufacturer's protocol.

**Cytokine Assays**

Cytokines were quantified via ELISA using a DuoSet kit (R&D Systems, Abingdon, United Kingdom), following the manufacturer's instructions. The minimum limits of detection were as follows: IL-1β 10 pg/mL; IL-6 10 pg/mL; IL-10 1.8 pg/mL; TNF-α 2.8 pg/mL; and IFN-γ 2.7 pg/mL.

**Curdlan Measurement**

Curdlan content was determined using the K-EBHLG Enzymatic Yeast b-Glucan Assay Kit (Megazyme, Bray, Ireland), with procedures specified by the manufacturer. Samples were lyophilized prior to analysis, and concentrations were expressed as a percentage of curdlan in the dry weight of the samples.

**PLF, BALF and Urine Cytology**

Fluid samples were collected in 200 µL K3-EDTA microtubes (Microvette, Sarstedt, Nümbrecht, Germany) and analyzed using the IDEXX ProCyte DxTM automated hematology instrument. The instrument employs flow cytometry with laser light scatter and cell staining to identify various cell types.

**Histological Evaluation and Wet-to-Dry Lung Weight Ratio**

Organ samples from the second phase of the experiment were preserved in 4% formalin for 24 hours, embedded in paraffin, and sectioned into 4µ sections for histological examination. Slides were stained with hematoxylin-eosin and examined using a light microscope. Additionally, kidney and liver samples were stained with periodic acid-Schiff reagent (PAS).
