# Supplementary material for: Long-Term Treatment with Alcaligenes faecalis A12C Improves Host Resistance to Pathogens in Septic Rats: Possible Contribution of Curdlan-Like Immune Trainer
Source: Probiotics Antimicrob Proteins. 2024 Apr 26;17(5):3100–19. doi: 10.1007/s12602-024-10252-0 (PMC12532692; doi:10.1007/s12602-024-10252-0)
Supplement: Supplementary file 3 — Supplementary file3 (DOCX 18 KB) [file 12602_2024_10252_MOESM3_ESM.docx]

SUPPLEMENTARY RESULTS

**Long-term treatment with *Alcaligenes faecalis* A12C improves host resistance to pathogens in septic rats: possible contribution of curdlan-like immune-trainer**

**^#1^C.J. Martel-Benítez** (ORCID iD: 0000-0002-1580-4058)

**^#1^R. Alayón-Afonso** (ORCID iD: 0000-0002-2928-0144)

**^*1^D.F. Padilla Castillo** (ORCID iD: 0000-0002-6678-5029)

**^2^F.J. Chamizo López** (ORCID iD: 0000-0003-1328-1924)

**^3,4^M. Isabel García-Laorden** (ORCID iD: 0000-0001-6270-6306)

**^5^ª. Espinosa de los Monteros y Zayas** (ORCID iD: 0000-0002-7736-3139)

**^6^J.C. Rivero-Vera** (ORCID iD: 0000-0003-3039-0030)

**^7^P. Nogueira Salgueiro** (ORCID iD: 0000-0002-8029-2685)

**^1^F. Real Valcárcel** (ORCID iD: 0000-0001-6526-0354)

**^2^ª. Bordes Benítez** (ORCID iD: 0000-0003-3243-7402)

^8^**ª. Martel Quintana** (ORCID iD: 0000-0002-7450-0505)

^9^**C. Almeida Peña** (ORCID iD: 0000-0001-9283-4851)

**^7^C. Domínguez Cabrera** (ORCID iD: 0000-0003-2600-1637)

**^10^J.M. González-Martín** (ORCID iD: 0000-0001-6816-4157)

**^11^J. Martín Caballero** (ORCID iD: 0000-0002-1579-2739)

**^12^R. Frías Beneyto** (ORCID iD: 0000-0001-7569-5693)

**^3,4^Jesús Villar** (ORCID iD: 0000-0001-5687-3562)

**^1,13,14,15^J.L. Martín-Barrasa,** (ORCID iD: 0000-0002-3280-9838)

From

*(1) Fish Health and Infectious Diseases Group, University Institute of Animal Health and Food Safety (IUSA), University of Las Palmas de Gran Canaria, Carretera de Trasmontana s/n, 35416 Arucas, Spain;*

*(2) Microbiology Department. Hospital Universitario de Gran Canaria Dr Negrín, Barranco de la Ballena s/n, 35019 Las Palmas de Gran Canaria, Spain;*

*(3) CIBER de Enfermedades Respiratorias, Instituto de Salud Carlos III, Monforte de Lemos 3-5, Pabellón 11, 28029 Madrid, Spain;*

*(4) Multidisciplinary Organ Dysfunction Evaluation Research Network, Research Unit, Hospital Universitario de Gran Canaria Dr. Negrín, Barranco de la Ballena s/n, 35019 Las Palmas de Gran Canaria, Spain;*

*(5) Morphology Department. University Institute of Animal Health and Food Safety (IUSA). Universidad de Las Palmas de Gran Canaria. Arucas. Las Palmas. Spain;*

*(6) Pathology Service. Hospital Universitario de Gran Canaria Dr Negrín, Barranco de la Ballena s/n, 35019 Las Palmas de Gran Canaria, Spain;*

*(7) Clinical Biochemistry Department. Hospital Universitario de Gran Canaria Dr Negrín, Barranco de la Ballena s/n, 35019 Las Palmas de Gran Canaria, Spain;*

*(8) Banco Español de Algas, Instituto de Oceanografía y Cambio Global, Universidad de Las Palmas de Gran Canaria, Telde, Spain;*

*(9) Banco Español de Algas, Fundación Parque Científico Tecnológico, Universidad de Las Palmas de Gran Canaria, Telde, Spain;*

*(10) Statistics Service. Research Unit, Hospital Universitario de Gran Canaria Dr Negrín, Barranco de la Ballena s/n, 35019 Las Palmas de Gran Canaria, Spain;*

*(11) Barcelona Biomedical Research Park (PRBB), Barcelona, Spain;*

*(12) Comparative Medicine, Karolinska Institutet, Stockholm, Sweden;*

*(13) Animal Facility, Research Unit, Hospital Universitario de Gran Canaria Dr Negrín, Barranco de la Ballena s/n, 35019 Las Palmas de Gran Canaria, Spain;*

*(14) Fundación Canaria del Instituto de Investigación Sanitaria de Canarias (FIISC), Las Palmas de Gran Canaria Spain;*

*(15) CIBER de Enfermedades Infecciosas (CIBERINFEC), Instituto de Salud Carlos III, Madrid, Spain.*

*#* Contributed equally

**∗Corresponding author:** DF Padilla Castillo**.** *Animal Infectious Diseases and Ictiopathology, University Institute of Animal Health and Food Safety, Universidad de Las Palmas de Gran Canaria, Carretera de Trasmontaña s/n, 35416 Arucas, Spain.* Phone: +(34)928459741. E-mail: daniel.padilla@ulpgc.es

**Supplementary Fig 1.** Body weight at different times in septic and healthy groups pretreated or no-pretreated with *A. faecalis* A12C.

The results are expressed as the body weight mean in grams and % of weight gained, assessed at 0 days (Initial) and 30 days (Pre CLP) of *A. faecalis* A12C or only water administration, and 20h Post CLP (AGUIC and AGUIA) or immediately before euthanasia (without CLP) in the case of healthy animals (AGUSAN and AGUSTO).
