# Supplementary material for: Long-Term Treatment with Alcaligenes faecalis A12C Improves Host Resistance to Pathogens in Septic Rats: Possible Contribution of Curdlan-Like Immune Trainer
Source: Probiotics Antimicrob Proteins. 2024 Apr 26;17(5):3100–19. doi: 10.1007/s12602-024-10252-0 (PMC12532692; doi:10.1007/s12602-024-10252-0)
Supplement: Supplementary file 4 — Supplementary file4 (DOCX 16 KB) [file 12602_2024_10252_MOESM4_ESM.docx]

**Supplementary Table 1.** Scoring of rodent protection test adapted from Acred et al. [24].

| **Vital sings** | |
| --- | --- |
| 1. Ruffled fur 2. Weight loss 3. Ocular discharge 4. Lethargy 5. Hunched posture | 1. Ataxia 2. Tremor 3. Hypothermia 4. Cyanosis |
| **Conditions** | **Suggested action** |
| 5+6 (or 7 or 8 or 9) | Euthanasia |
