# Supplementary material for: Long-Term Treatment with Alcaligenes faecalis A12C Improves Host Resistance to Pathogens in Septic Rats: Possible Contribution of Curdlan-Like Immune Trainer
Source: Probiotics Antimicrob Proteins. 2024 Apr 26;17(5):3100–19. doi: 10.1007/s12602-024-10252-0 (PMC12532692; doi:10.1007/s12602-024-10252-0)
Supplement: Supplementary file 5 — Supplementary file5 (DOCX 17 KB) [file 12602_2024_10252_MOESM5_ESM.docx]

|  | **AGUSAN** | **AGUSTO** | **AGUIC** | **AGUIA** |
| --- | --- | --- | --- | --- |
| **RBC (x10^6^/µL)** | 10.41  (10.32 – 10.48) | 11.05  (10.74-11.14) | 10.41  (10.25 – 11.53) | 10.58  (10.25 -10.83) |
| **HGB (g/dL)** | 17.60  (17.50 – 17.80) | 18.20  (17.50-19.10) | 17.55  (16.88 – 19.08) | 17.75  (17.38 – 18.28) |
| **HCT(%)** | 53.60  (52.50 – 55.00) | 54.5  (52.90-57.50) | 50.10  (49.43 – 54.45) | 52.40  (50.93 – 53.80) |
| **PLT (x10^3^/µL)** | 900.00  (843.00 – 957.00) | 839.00  (790.00-869.00) | 442.50  (300.50 – 575.25) | 558.50  (528.50 – 605.50) |
| **WBC (x10^3^/µL)** | 5.54  (5.17 – 5.72) | 7.22  (6.69-7.37) | 1.89  (1.52 – 4.02) | 4.02  (3.19 – 4.59) |
| **N (%)** | 17.00  (15.80 – 20.70) | 14.20  (13.00-15.20) | 8.55  (7.03 – 12.00) | 9.30  (6.83 – 10.88) |
| **M (%)** | 6.90  (1.90 – 7.20) | 8.20  (7.70-8.90) | 26.35  (20.23 – 28.75) | 30.40  (29.18 – 31.78) |
| **E (%)** | 1.80  (1.60 – 2.10) | 1.30  (1.00-1.50) | 0.65  (0.50 – 0.80) | 0.40  (0.30 – 0.58) |
| **B (%)** | 0.20  (0.20 – 0.30) | 0.20  (0.10-0.30) | 0.30  (0.00 – 0.50) | 0.20  (0.00 – 0.38) |
| **L (%)** | 75.50  (71.40 – 78.30) | 76.50  (74.70-79.40) | 64.75  (60.43 – 67.98) | 60.00  (58.20 – 63.68) |

**Supplementary Table 2**. Hematological parameters at the end of the experimental phase 2 (20h after CLP) in all groups

Results are expressed as the median (P25–P75) from rats before euthanasia

RBC total number of erythrocytes, HGB haemoglobin concentration, HCT Hematocrit value: erythrocyte ratio of total blood volume, PLT total number of platelets, WBC total number of leukocytes, S% segmented neutrophil percent, M% monocyte percent, E% eosinophil percent, B% basophil percent, L% lymphocyte percent

** P < 0.01
