# Supplementary material for: Long-Term Treatment with Alcaligenes faecalis A12C Improves Host Resistance to Pathogens in Septic Rats: Possible Contribution of Curdlan-Like Immune Trainer
Source: Probiotics Antimicrob Proteins. 2024 Apr 26;17(5):3100–19. doi: 10.1007/s12602-024-10252-0 (PMC12532692; doi:10.1007/s12602-024-10252-0)
Supplement: Supplementary file 6 — Supplementary file6 (DOCX 23 KB) [file 12602_2024_10252_MOESM6_ESM.docx]

| **Ref** | **Blood** | **BALF** | **PLF** | **Urine** |
| --- | --- | --- | --- | --- |
| **AGUSAN-1** | *-* | *-* | *-* | *-* |
| **AGUSAN-2** | *-* | *-* | *-* | *-* |
| **AGUSAN-3** | *-* | *-* | *-* | *-* |
| **AGUSAN-4** | *-* | *-* | *-* | *-* |
| **AGUSAN-5** | *-* | *-* | *-* | *-* |
| **AGUSAN-6** | *-* | *-* | *-* | *-* |
| **AGUSAN-7** | *-* | *Muribacterium muris* | *-* | *-* |
| **AGUSAN-8** | *-* | *-* | *-* | *-* |
| **AGUSAN-9** | *-* | *Muribacterium muris* | *-* | *-* |
| **AGUSTO-1** | *-* | *-* | *-* | *-* |
| **AGUSTO-2** | *-* | *-* | *-* | *-* |
| **AGUSTO-3** | *-* | *-* | *-* | *-* |
| **AGUSTO-4** | *-* | *Muribacterium muris* | *-* | *-* |
| **AGUSTO-5** | *-* | *Klebsiella pneumoniae* | *-* | *-* |
| **AGUSTO-6** | *-* | *-* | *-* | *-* |
| **AGUSTO-7** | *-* | *Corynebacterium striatum*  *Lactobacillus paracasei*  *Staphylococcus (coag -)* | *-* | *-* |
| **AGUSTO-8** | **-** | **-** | **-** | **-** |
| **AGUSTO-9** | *-* | *-* | *-* | *-* |
| **AGUIC-1** | *E. coli* | *E. coli* | *E. coli*  *Bacteroides uniformis* | *E.coli* |
| **AGUIC-2** | *E. coli* | *E. coli* | *E. coli* | *E. coli* |
| **AGUIC-3** | *E. coli* | *E. coli* | *E.coli*  *Staph. sciuri* | *E. coli* |
| **AGUIC-4** | *E. coli* | *E. coli* | *E. coli*  *Enterococcus gallinarum* | *E. coli* |
| **AGUIC-5** | *E. coli* | *E. coli*  *Rodentibacter pneumotropicus*  *Str. lentus* | *E. coli*  *Enterococcus gallinarum* | *-* |
| **AGUIC-6** | *E. coli* | *E. coli* | *E. coli*  *Bacteroides uniformis* | *E. coli* |
| **AGUIC-7** | *E. coli* | *-* | *E. coli*  *L. murinus* | *-* |
| **AGUIC-8** | *E. coli* | *-* | *E. coli* | *Acinetobacter iwoffi* |
| **AGUIC-9** | *E. coli* | *E. coli*  *Str. cohnii* | *E. coli*  *Myroides odoratimimus* | *E. coli* |
| **AGUIC-10** | *E. coli*  *L. murinus* | *-* | *E. coli* | *E. coli* |
| **AGUIC-11** | *E. coli* | *E. coli* | *E. coli* | *E. coli* |
| **AGUIC-12** | *E. coli* | *Str. viridans*  *E. coli*  *Moraxella osloensis*  *Rodentibacter pneumotropicus*  *Str. mitis* | *E. coli*  *Enterococcus gallinarum* | *E. coli* |
| **AGUIC-13** | *E. coli* | *E. coli* | *-* | *E. coli* |
| **AGUIA-1** | *E.coli* | *-* | *Stph. epidermidis*  *L. murinus*  *E.coli* | *-* |
| **AGUIA-2** | *E.coli* | *E.coli*  *Str. lentus* | *E.coli*  *Str. lentus*  *Corynebacterium striatum*  *L. paracasei* | *-* |
| **AGUIA-3** | *E.coli* | *E.coli* | *E.coli*  *Str. homini* | *-* |
| **AGUIA-4** | *Bacteroides uniformis* | *Str. Sciuri* | *L. coryniformis* | *-* |
| **AGUIA-5** | - | *-* | *L. murinus*  *Str. capitis*  *Enterococcus faecalis* | *-* |
| **AGUIA-6** | *-* | *E.coli*  *Haemophylus ahemolyticus* | *E.coli* | *-* |
| **AGUIA-7** | *E.coli* | *-* | *E.coli*  *L. murinus*  *Staph. sciuri*  *Enterococcus faecalis*  *A. faecalis* | *Lactobacillus spp.* |
| **AGUIA-8** | *E.coli* | *-* | *E.coli*  *Cutibacterium acnes*  *Bacteroides uniformis* | *-* |
| **AGUIA-9** | *E.coli*  *Lac. murinus* | *-* | *E.coli* | *-* |
| **AGUIA-10** | *E.coli*  *Lac. murinus* | *E.coli* | *E.coli*  *Alcaligenes faecalis* | *E.coli* |
| **AGUIA-11** | *E.coli*  *Str. suis* | *-* | *E.coli*  *Bacteroides uniformis* | *-* |
| **AGUIA-12** | *E.coli* | *E.coli* | *E.coli*  *L. murinus*  *Enterococcus faecalis* | *-* |
| **AGUIA-13** | *E.coli* | *-* | *E. coli*  *Enterococcus faecalis* | *-* |
| **AGUIA-14** | *E.coli* | *E.coli* | *E.coli* | *-* |

**Supplementary Table 3**. Microbiological study 20h after CLP in all groups (phase 2).
