# Supplementary material for: Long-Term Treatment with Alcaligenes faecalis A12C Improves Host Resistance to Pathogens in Septic Rats: Possible Contribution of Curdlan-Like Immune Trainer
Source: Probiotics Antimicrob Proteins. 2024 Apr 26;17(5):3100–19. doi: 10.1007/s12602-024-10252-0 (PMC12532692; doi:10.1007/s12602-024-10252-0)
Supplement: Supplementary file 7 — Supplementary file7 (DOCX 23 KB) [file 12602_2024_10252_MOESM7_ESM.docx]

|  | **AGUSAN** | **AGUSTO** | **AGUIC** | **AGUIA** |  |
| --- | --- | --- | --- | --- | --- |
|  | **PSN=0** | **PSN=0** | **PSN=13** | **PSN=12** |  |
| ***Escherichia coli*** |  |  | 92.9% | 73.3% | Blood |
| ***Lactobacillus murinus*** |  |  | 7.1% | 13.3% |  |
| ***Bacteroides uniformis*** |  |  |  | 6.7% |  |
| ***Streptococcus suis*** |  |  |  | 6.7% |  |
|  | **PSN=0** | **PSN=0** | **PSN=12** | **PSN=14** |  |
| ***Escherichia coli*** |  |  | 60% | 36.4% | PLF |
| ***Enterococcus gallinarum*** |  |  | 15% |  |  |
| ***Bacteroides uniformis*** |  |  | 10% | 6.1% |  |
| ***Staphylococcus sciuri*** |  |  | 5% | 3% |  |
| ***Lactobacillus murinus*** |  |  | 5% | 12.1% |  |
| ***Myroides odoratimimus*** |  |  | 5% |  |  |
| ***Staphylococcus epidermidis*** |  |  |  | 3% |  |
| ***Staphylococcus lentus*** |  |  |  | 3% |  |
| ***Corynebacterium striatum*** |  |  |  | 3% |  |
| ***Lactobacillus paracasei*** |  |  |  | 3% |  |
| ***Staphylococcus homini*** |  |  |  | 3% |  |
| ***Lactobacillus coryniformis*** |  |  |  | 3% |  |
| ***Staphylococcus capitis*** |  |  |  | 3% |  |
| ***Enterococcus faecalis*** |  |  |  | 12.1% |  |
| ***Alcaligenes faecalis*** |  |  |  | 6.1% |  |
| ***Cutibacterium acnes*** |  |  |  | 3% |  |
|  | **PSN=2** | **PSN=3** | **PSN=10** | **PSN=7** |  |
| ***Escherichia coli*** |  |  | 58.8% | 66.7% | BALF |
| ***Muribacterium muris*** | 100% | 20% |  |  |  |
| ***Klebsiella pneumoniae*** |  | 20% |  |  |  |
| ***Corynebacterium striatum*** |  | 20% |  |  |  |
| ***Lactobacillus paracasei*** |  | 20% |  |  |  |
| ***Staphylococcus (coag -)*** |  | 20% |  |  |  |
| ***Rodentibacter pneumotropicus*** |  |  | 11.8% |  |  |
| ***Staphylococcus lentus*** |  |  | 5.9% | 11.1% |  |
| ***Staphylococcus cohnii*** |  |  | 5.9% |  |  |
| ***Streptococcus viridans*** |  |  | 5.9% |  |  |
| ***Moraxella osloensis*** |  |  | 5.9% |  |  |
| ***Streptococcus mitis*** |  |  | 5.9% |  |  |
| ***Staphylococcus sciuri*** |  |  |  | 11.1% |  |
| ***Haemophilus haemolyticus*** |  |  |  | 11.1% |  |
|  | **PSN=0** | **PSN=0** | **PSN=11** | **PSN=2** |  |
| ***Escherichia coli*** |  |  | 90.9% | 50% | Urine |
| ***Acinetobacter iwoffi*** |  |  | 9.1% |  |  |
| ***Lactobacillus spp.*** |  |  |  | 50% |  |

**Supplementary Table 4**. Samples culture analysis at the end of the experimental phase 2 in all experimental groups

Results are expressed as percentages (%) of positive samples number (PSN) in each experimental groups
